# Patient-specific computational simulation of coronary artery bifurcation stenting

**DOI:** 10.1038/s41598-021-95026-2

**Published:** 2021-08-13

**Authors:** Shijia Zhao, Wei Wu, Saurabhi Samant, Behram Khan, Ghassan S. Kassab, Yusuke Watanabe, Yoshinobu Murasato, Mohammadali Sharzehee, Janaki Makadia, Daniel Zolty, Anastasios Panagopoulos, Francesco Burzotta, Francesco Migliavacca, Thomas W. Johnson, Thierry Lefevre, Jens Flensted Lassen, Emmanouil S. Brilakis, Deepak L. Bhatt, George Dangas, Claudio Chiastra, Goran Stankovic, Yves Louvard, Yiannis S. Chatzizisis

**Affiliations:** 1 Cardiovascular Biology and Biomechanics Laboratory, Cardiovascular Division, University of Nebraska Medical Center, Omaha, NE USA; 2 California Medical Innovation Institute, San Diego, CA USA; 3 Department of Cardiology, Teikyo University Hospital, Tokyo, Japan; 4 Department of Cardiology, National Hospital Organization Kyushu Medical Center, Fukuoka, Japan; 5 Department of Cardiovascular Sciences, Fondazione Policlinico Universitario A. Gemelli IRCCS Università Cattolica del Sacro Cuore, Rome, Italy; 6 Laboratory of Biological Structure Mechanics (LaBS), Department of Chemistry, Materials and Chemical Engineering “Giulio Natta”, Politecnico di Milano, Milan, Italy; 7 Department of Cardiology, Bristol Heart Institute, University Hospitals Bristol NHSFT and University of Bristol, Bristol, UK; 8 Ramsay Générale de Santé - Institut cardiovasculaire Paris Sud, Hopital Privé Jacques Cartier, Massy, France; 9 Department of Cardiology B, Odense Universitets Hospital and University of Southern Denmark, Odense C, Denmark; 10 Minneapolis Heart Institute, Minneapolis, MN USA; 11 Brigham and Women’s Hospital, Harvard Medical School, Boston, MA USA; 12 The Zena and Michael A. Wiener Cardiovascular Institute, Mount Sinai Hospital, Icahn School of Medicine, New York City, NY USA; 13 PoliTo(BIO)Med Lab, Department of Mechanical and Aerospace Engineering, Politecnico di Torino, Turin, Italy; 14 Department of Cardiology, Clinical Center of Serbia, Belgrade, Serbia; 15 Institut Cardiovasculaire Paris Sud, Massy, France

**Keywords:** Interventional cardiology, Biomedical engineering

## Abstract

Patient-specific and lesion-specific computational simulation of bifurcation stenting is an attractive approach to achieve individualized pre-procedural planning that could improve outcomes. The objectives of this work were to describe and validate a novel platform for fully computational patient-specific coronary bifurcation stenting. Our computational stent simulation platform was trained using n = 4 patient-specific bench bifurcation models (n = 17 simulations), and n = 5 clinical bifurcation cases (training group, n = 23 simulations). The platform was blindly tested in n = 5 clinical bifurcation cases (testing group, n = 29 simulations). A variety of stent platforms and stent techniques with 1- or 2-stents was used. Post-stenting imaging with micro-computed tomography (μCT) for bench group and optical coherence tomography (OCT) for clinical groups were used as reference for the training and testing of computational coronary bifurcation stenting. There was a very high agreement for mean lumen diameter (MLD) between stent simulations and post-stenting μCT in bench cases yielding an overall bias of 0.03 (− 0.28 to 0.34) mm. Similarly, there was a high agreement for MLD between stent simulation and OCT in clinical training group [bias 0.08 (− 0.24 to 0.41) mm], and clinical testing group [bias 0.08 (− 0.29 to 0.46) mm]. Quantitatively and qualitatively stent size and shape in computational stenting was in high agreement with clinical cases, yielding an overall bias of < 0.15 mm. Patient-specific computational stenting of coronary bifurcations is a feasible and accurate approach. Future clinical studies are warranted to investigate the ability of computational stenting simulations to guide decision-making in the cardiac catheterization laboratory and improve clinical outcomes.

## Introduction

Coronary bifurcations remain one of the most challenging lesion subsets in interventional cardiology with lower procedural success rates than non-bifurcations, and increased rates of adverse cardiac events, ranging between 15 and 20% at 6 months to 1 year post intervention^1^. The stenting technique and the associated biomechanical environment play a dominant role in the restenosis propensity of this type of lesions^1–3^. Consideration of patient-specific anatomic parameters and local physiologic/biomechanical factors has the potential to optimize bifurcation stenting.

In the era of powerful computers, patient-specific computational simulation of bifurcation stenting can provide individualized pre-procedural planning and improvement of outcomes^1^. Computational simulations could guide percutaneous interventions with incremental information to the anatomical and functional assessment of coronary artery disease in the catheterization laboratory^4^. Computational stenting models can reproduce controversial “what if” scenarios in a 3D environment and in a cost- and time-effective fashion, elucidating the events occurring during the stenting procedure^5^. Computational stenting can characterize the local biomechanical microenvironment pre- and post-stenting, providing a framework for bifurcation stenting optimization and generating new hypotheses that can be tested clinically.

To date, several computational stent simulation studies have been reported, with the majority focusing on idealized non-bifurcated geometries^6–10^. Very few computational studies have focused on coronary bifurcations, mostly simulating one-stent techniques using simplified plaque material properties^11,12^. In this work, we aimed to present and validate a novel platform for fully computational, patient-specific coronary bifurcation stenting. Our computational simulation platform was trained in patient-specific bench bifurcation models and clinical cases. We blindly validated our platform in clinical cases. A variety of stent techniques and stent platforms were used.

## Materials and methods

The study design of bench and clinical testing is summarized in the Online Figure 1.

### Patient-specific bench stenting

#### Silicone bifurcation models

Four bench models of patient-specific coronary artery bifurcations were created using an in-house developed technique^13^. The initial bifurcation geometries were 3D reconstructed from human coronary angiograms (CAAS, Pie Medical Imaging BV, Maastricht, The Netherlands). For each model, a negative mold was designed, and 3D printed with acrylonitrile butadiene styrene material. After smoothing the inner surface of the mold using acetone vapor, polydimethylsiloxane (Sylgard 184, Dow Corning Corporation, Midland, MI, USA) was injected into the mold and then placed in an oven for curing of polydimethylsiloxane. The physical models were then immersed in an acetone beaker to dissolve the acrylonitrile butadiene styrene and generate the final silicone bifurcation models for bench testing.

#### Contrast enhanced micro-computed tomography (μCT) imaging

To acquire high-resolution lumen geometry, all silicone bifurcation models were filled with contrast and scanned with μCT (Bruker SkyScan 1172, Kontich, Belgium, https://www.bruker.com/ru/products-and-solutions/preclinical-imaging/micro-ct/skyscan-1272.html) using the following parameters: Image pixel size (26.9 μm), voltage (100 kV), and current (100 μA). The reconstructed 3D models based on μCT before stenting served as anatomical input to computational stent simulations^13^.

#### Bench stenting of silicone bifurcation models

The silicone bifurcation models were placed in a custom-made flow chamber^13^. A computer-controlled bioreactor circuit was connected to the inlet and outlet of the bifurcation and facilitated the circulation of 1 L of deionized water at a steady flow-rate of 100 mL/min. The stenting techniques performed on the silicone bifurcation models are summarized in Table 1.

**Table 1:** Stenting techniques and procedural steps in bench cases.

Step	Bench #1 (Provisional)	Bench #2 (TAP with long protrusion)	Bench #3 (Culotte)	Bench #4 (TAP)
#1	MV stenting: Resolute Integrity 3.0 × 26 mm@16 atm	MV stenting: Resolute Integrity 3.5 × 22 mm@14 atm	SB stenting: Resolute Onyx 3.0 × 18 mm@12 atm	MV stenting: Synergy 4.0 × 16 mm@14 atm
#2	POT: Compliant balloon 3.5 × 15 mm@16 atm	POT: Compliant balloon 3.5 × 15 mm@16 atm	1st POT: Compliant balloon 3.0 × 15 mm@10 atm	SB stenting: Synergy 3.0 × 16 mm@12 atm
#3		SB strut opening: Compliant balloon 3.0 × 15 mm@14 atm	2nd POT: Compliant balloon 3.0 × 15 mm@14 atm	KBI: Compliant balloon 4.0 × 15 mm@10 atm in MV and stent balloon 3.0 × 16 mm@12 atm in SB
#4		SB stenting: Resolute Integrity 2.75 × 8 mm@18 atm (with long protrusion into MV)	MV strut opening: Compliant balloon 3.0 × 15 mm@6 atm	
#5		KBI: Compliant balloon 3.5 × 15 mm@12 atm in MV and stent balloon 2.75 × 8 mm@14 atm in SB	MV stenting: Resolute Onyx 3.0 × 18 mm@12 atm	
#6			3rd POT: Compliant balloon 3.0 × 15 mm@16 atm	
#7			KBI: Compliant balloon 3.0 × 15 mm@14 atm in MV and Compliant balloon 3.0 × 15 mm@14 atm in SB	

*MV* main vessel, *SB* side branch, *POT* proximal optimization technique, *KBI* kissing balloon inflation, *TAP* T-and Protrusion technique, *atm* atmosphere.

#### Stereoscopic scanning

All the stents deployed in the silicone bifurcation models were imaged with a stereoscopic microscope (Olympus SZX16, Olympus Corporation, Tokyo, Japan). The microscopic images were used to measure the distance of the stent edges from fixed points (e.g. carina) and guide stent positioning in the computational models.

#### Computational simulation of bench bifurcation stenting

*Computational mesh* The 3D reconstructed lumens by μCT were meshed with four-node quadrilateral shell elements using HyperMesh (Altair Engineering, Troy, MI, USA). The stent design models used in bench stenting were provided by the manufacturers in their nominal dimensions. The balloons were computationally created in Grasshopper (plugin to Rhinoceros 6.0, Robert McNeel and Associates, Seattle, WA, USA) in their crimped state. The stents were meshed in HyperMesh using beam elements (Resolute Integrity and Onyx; Medtronic Vascular, Santa Rosa, CA, USA) or hexahedral elements (Synergy; Boston Scientific, Maple Groove, MN, USA), whereas the balloons were meshed with quadrilateral finite-membrane-strain elements.

*Material properties* The stent and silicone material properties used in the computational simulations of bench models are listed in Table 2^14^. The cured silicone samples (“Silicone bifurcation models” section) were cut into rectangular specimens and underwent uni-axial compression testing. The obtained force–displacement curves were converted into stress–strain curves. The Neo-Hookean hyperelastic model was used to fit the non-linear stress–strain curve. A specific thickness was assigned to the shell elements of each bifurcation to represent the true thickness of the silicone models. The elastic modulus for compliant, semi- and non-compliant balloons was defined as 300 MPa, 900 MPa and 1500 MPa, respectively^15^.

**Table 2:** Coefficients for the material models used in computational stenting simulations of bench and clinical cases.

	C10 (MPa)	C20 (MPa)	C30 (MPa)	C40 (MPa)	C50 (MPa)	C60 (MPa)	Yield stress (MPa)	Density (g/cm^3^)
Silicone	0.154	–	–	–	–	–	–	2.32
Normal wall^20^	6.52e−3	4.89e−2	9.26e−3	0.76	− 0.43	8.69e−2	–	
Very soft	0.045	0.17	− 0.13	0.11	–	–	0.12	
Soft	0.01	0.49	4.13	–	–	–	0.71	
Neutral	0.06	4.28	− 21.36	69.36	–	–	1.37	
Stiff	0.11	9.06	–	–	–	–	1.81	
Very stiff	0.21	64.86	− 3.5e3	1.999e5	–	–	627	

	Elastic modulus (GPa)	0.2% Yield strength (MPa)	Tensile strength (MPa)	Elongation (%)				
MP35N^14^ (Resolute Integrity and Onyx)	233	414	930	45				8.40
Pt–Ir^32^ (Resolute Onyx)	224	285	–	–				21.6
Pt–Cr^14^ (Synergy)	203	480	834	45				9.90

*Stent and balloon crimping, positioning, and bending* The correct stent and balloon positioning in the computational bifurcation models was determined by angiography, μCT and stereoscopic imaging of the stented silicone models. The stents were first crimped from their nominal states using surface elements driven by radial displacement. Then, the crimped stents and balloons were positioned and bent along the artery centerline^16^.

*Computational simulation of bench stenting procedures* The bench stenting procedures were simulated through a multi-step, quasi-static finite element analysis using the central difference method (Abaqus/Explicit solver, Dassault Systèmes Simulia Corporation, Providence, RI, USA). The steps followed in the stenting procedures are summarized in Table 1. Only the edges of the bifurcation lumen were fixed to avoid rigid body motion. Balloon edges were constrained to eliminate motion in all directions. To model the interactions between different elements (balloon–stent, stent–lumen, balloon–lumen, balloon–balloon, stent–stent), the robust general contact algorithm was used with a friction coefficient of 0.2^17^. The real inflation pressures used in each procedural step were applied to the inner surface of the corresponding balloons. The stressed configurations of lumen and stent after each step were used as the initial condition for the next step. Given the large number of elements and complicated contacts in the computational model, a computer cluster (452 Intel Xeon E5-2670 2.60 GHz 2 CPU/16 cores and 64 GB RAM per node, University of Nebraska, NE, USA) was used to perform the high-speed computational simulations.

#### Training of computational bench stenting

The 3D reconstructed bifurcation and stent geometry by μCT post-stenting served as the ground truth for the training of computational stenting. The simulated bifurcation and μCT bifurcation were co-registered using the bifurcation carina as the fixed point. The mean lumen diameter (MLD) was used for the comparison studies.

### Clinical stenting

#### Patient data

Ten patient cases were selected for the patient-specific computational simulations from PROPOT (Randomized trial of the proximal optimization technique in coronary bifurcation lesions), a multi-center, prospective, open-label study that compared proximal optimization technique (POT) versus kissing balloon inflation (KBI) in provisional stenting of coronary bifurcations using Zotarolimus-eluting stents (Resolute Integrity or Onyx, Medtronic; Online Table 1). The use of these geometries was approved by the ethics committee of Teikyo University (IRB approval number #15-159-2). All methods were carried out in accordance with relevant guidelines and regulations, and informed consent was obtained from all subjects.

**Figure 1:**
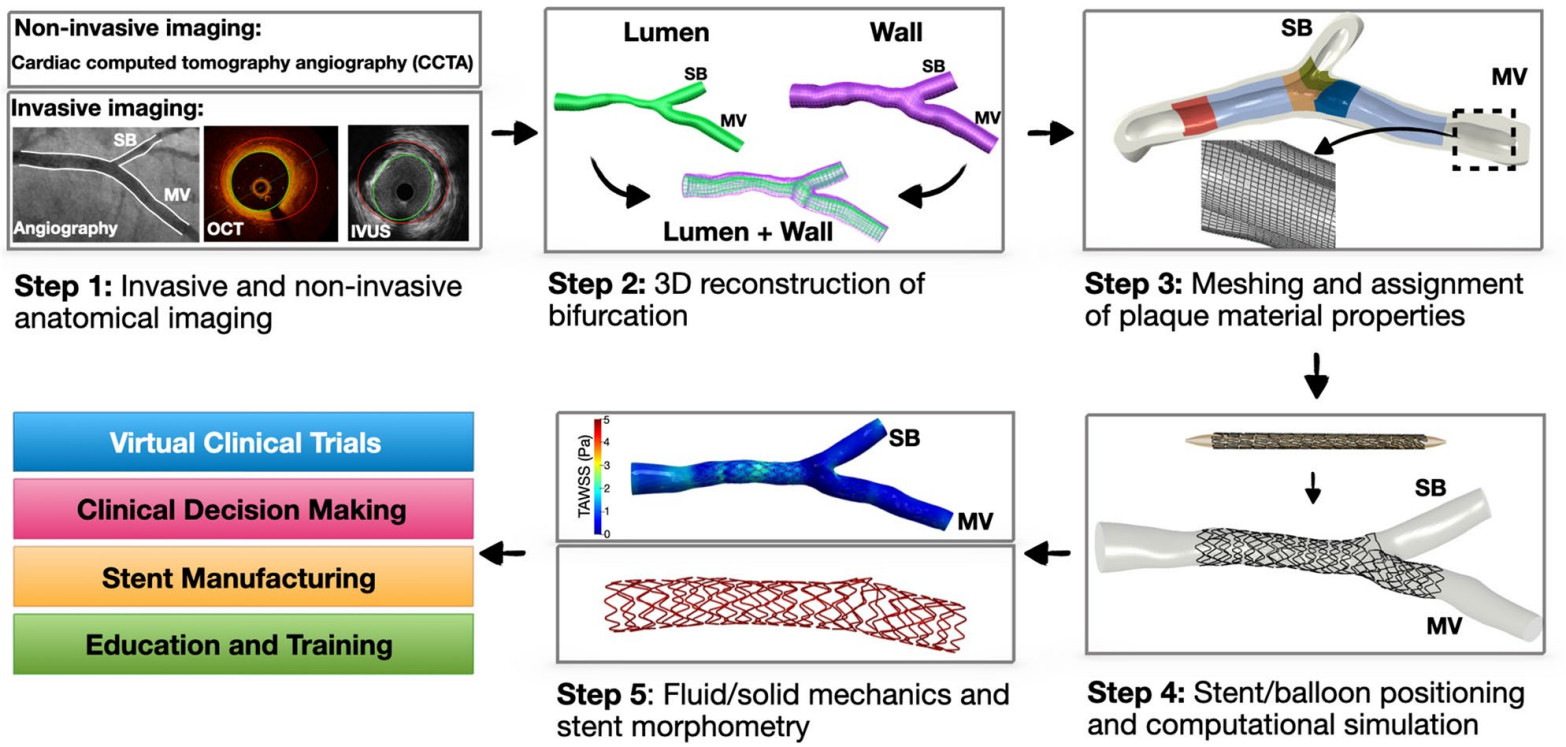
Workflow of patient-specific computational stenting simulations. Overview of the steps required for computational simulations of bifurcation stenting. The bifurcation lumen and wall are 3D reconstructed by fusing angiography and OCT (Steps 1 and 2). The 3D reconstructed model is meshed and assigned with patient-specific plaque material properties based on OCT imaging (Step 3). The real stent and balloon designs are computationally positioned in the bifurcation (Step 4). Using finite element method, the stenting procedure is computationally performed (Step 4). The output of the simulation includes stent morphometry and biomechanics (Step 5); *IVUS* intravascular ultrasound, *OCT* optical coherence tomography, *SB* side branch, *MV* main vessel, *TAWSS* time-averaged wall shear stress.

All patients of this study underwent coronary angiography (at multiple angiographic planes) and intracoronary imaging with optical coherence tomography (OCT) of main vessel (MV) and side branch (SB) before the percutaneous coronary intervention (PCI), immediately post stenting, and at the end of the procedure. Pre-PCI anatomical imaging data were used to 3D reconstruct the patient-specific coronary bifurcation anatomies which served as anatomical input to the computational stenting simulations^13^. The steps of computational simulations of clinical bifurcation stenting is illustrated in Fig. 1.

*Training group* Five out of ten cases (patient #1–5, Table 3) were used for training of the computational stenting platform, wherein none of the operators were blinded. All the PCI steps performed in this group of cases were replicated in the computational environment as described in “Computational simulation of clinical stenting” section. The post-PCI OCT data were used as the ground truth for the comparison to the computational simulations.

**Table 3:** Bifurcation stenting steps in clinical cases.

**I. Training Group**					
Step	Patient #1	Patient #2	Patient #3	Patient #4	Patient #5
#1	MV stenting Resolute integrity 3.5 × 18 mm@8 atm	MV pre-dilatation SC balloon 2.5 × 15 mm@14 atm	MV stenting Resolute integrity 3.0 × 38 mm@12 atm	MV pre-dilatation Compliant balloon 2 × 20 mm@8 atm	MV stenting Resolute Onyx 2.5 × 18 mm@18 atm
#2	1st POT NC balloon 3.75 × 8 mm@18 atm	MV stenting Resolute integrity 2.5 × 18 mm@12 atm	1st POT NC balloon 3.5 × 6 mm@18 atm	MV stenting Resolute Onyx 3 × 22 mm@14 atm	1st POT NC balloon 3.5 × 8 mm@18 atm
#3	2nd POT NC balloon 3.75 × 8 mm@18 atm	KBI SC balloon 2.5 × 15 mm@14 atm in MV and SC balloon 2 × 15 mm@8 atm in SB	2nd POT NC balloon 3.5 × 6 mm@18 atm, more proximally than step #2	1st POT Stent balloon 3 × 22 mm@14 atm more proximally than step #2	2nd POT NC balloon 3.5 × 8 mm@18 atm, more proximally than step #2
#4	SB strut opening SC balloon 2.5 × 4 mm@6 atm		3rd POT NC balloon 3.5 × 6 mm@18 atm, more proximally than step #3	SB strut opening Compliant balloon 2.5 × 20 mm@6 atm	SB strut opening NC balloon 2.25 × 12 mm@14 atm
#5			SB strut opening Compliant balloon 1.5 × 15 mm@10 atm	1st KBI NC balloon 2.75 × 15 mm@6 atm in MV and compliant balloon 2.5 × 20 mm@6 atm in SB	3rd POT NC balloon 3.5 × 8 mm@18 atm, more proximally than step #3
#6				2nd KBI NC balloon 2.75 × 15 mm@6 atm in MV more proximally than step #5 and compliant balloon 2.5 × 20 mm@6 atm in SB	

**II. Testing Group**					
Step	Patient #6	Patient #7	Patient #8	Patient #9	Patient #10
#1	1st MV pre-dilatation NC balloon 3.0 × 12 mm@16 atm	MV pre-dilatation SC balloon 2.5 × 15 mm@6 atm	MV stenting Resolute Integrity 2.75 × 22 mm@10 atm	Pre-dilatation SC balloon 3.0 × 15 mm@14 atm	MV stenting Resolute Onyx 3.0 × 18 mm@12 atm
#2	2nd MV pre-dilatation NC balloon 3.0 × 12 mm@16 atm more distally than step #1	MV stenting Resolute Integrity 2.5 × 18 mm@16 atm	SB strut opening NC balloon 2.25 × 12 mm@8 atm	MV stenting Resolute Onyx 3.5 × 18 mm@12 atm	SB strut opening NC balloon 2.0 × 12 mm@14 atm
#3	3rd MV pre-dilatation NC balloon 3.0 × 12 mm@16 atm more proximally than step #1	1st POT Stent balloon 2.5 × 18 mm@16 atm more proximally than step #2	POT NC balloon 2.5 × 12 mm@12 atm	1st POT NC balloon 3.75 × 8 mm@14 atm	Post-dilatation Stent balloon 3.0 × 18 mm@12 atm
#4	MV stenting Resolute Integrity 3.0 × 38 mm@12 atm	2nd POT NC balloon 3.0 × 8 mm@14 atm	1st KBI NC balloon 2.5 × 12 mm@12 atm in MV and NC balloon 2.25 × 12 mm@8 atm in SB	2nd POT NC balloon 3.75 × 8 mm@14 atm, more proximally than step #3	1st KBI SC balloon 3.0 × 18 mm@12 atm in MV and NC balloon 2.0 × 12 mm@14 atm in SB
#5	SB strut opening SC balloon 2.0 × 15 mm@6 atm	3rd POT NC balloon 3.0 × 8 mm@14 atm more proximally than step #4	2nd KBI NC balloon 2.5 × 12 mm@12 atm in MV and NC balloon 2.25 × 12 mm@8 atm in SB more proximally than step #4	SB strut opening NC balloon 2.25 × 8 mm@12 atm	2nd KBI SC balloon 3.0 × 18 mm@12 atm in MV and NC balloon 2.0 × 12 mm@14 atm in SB more proximally than step #4
#6	KBI NC balloon 3.0 × 12 mm@6 atm in MV and SC balloon 2.0 × 15 mm@6 atm in SB	4th POT NC balloon 3.0 × 8 mm@14 atm more proximally than step #5			
#7	POT NC balloon 3.0 × 12 mm@6 atm	SB strut opening SC balloon 2.0 × 4 mm@6 atm			

*MV* main vessel, *SB* side branch, *POT* proximal optimization technique, *KBI* kissing balloon inflation, *NC* non-compliant, *SC* semi-compliant, *atm* atmosphere.

*Testing group* Five cases (patient #6–10, Table 3**)** were chosen for testing of the computational stenting platform. The operators responsible for angiography and OCT imaging analysis, 3D reconstruction of vessels, computational stenting simulations and comparison studies were blinded to each other. The stenting simulation results were compared with the post-PCI OCT data.

#### 3D reconstruction of bifurcation geometry

The pre-PCI bifurcation geometries were 3D reconstructed from fusion of angiography with OCT^13^. The bifurcation centerline was generated from two angiographic planes (CAAS, Pie Medical Imaging BV, Maastricht, The Netherlands), and served as the backbone for the reconstruction. The segmented OCT images (EchoPlaque 4.0, INDEC Medical System, Los Altos, CA, USA) were aligned along the centerline using carina as reference point. We followed a systematic approach for the delineation of the outer borders in OCT images, as previously described^13^. In cases of ill-defined outer wall borders, our approach involved limiting the segmentation at the margin of complete signal loss. If the margin of complete signal loss could not be identified in < 180 degrees of vessel circumference, we interpolated with the visible border, but if it was unidentifiable in > 180 degrees of vessel circumference, we discarded that particular OCT frame. The aligned lumen and wall contours were lofted to build the MV and SB inner and outer surfaces. Then, the MV and SB surfaces were merged to create the bifurcation lumen and wall.

#### Computational simulation of clinical stenting

*Computational mesh* The 3D reconstructed bifurcation models were meshed with 0.25 mm hexahedral elements (ICEM CFD 17.2, ANSYS Inc., Canonsburg, PA, USA). Mesh convergence study using different element sizes (from 0.50 to 0.25 mm) showed minimal difference (< 1%) in stent expansion^18^. The stent design models were provided by the manufacturer (Medtronic Vascular) in their nominal dimensions. The balloons were constructed in Grasshopper in their crimped state. The stents and balloons were meshed using fully hexahedral and quadrilateral finite-membrane-strain elements, respectively.

*Material properties* In computational simulations, the wall thickness, lumen area, plaque eccentricity and plaque material were determined by OCT, as previously described^18^. A novel plaque scoring system was established based on the experimental data represented by the stress-strain graph of the constitutive equation with multiple coefficients (Online Figure 2)^18,19^. The area, circumference, and thickness of the lipid, fibrous, and calcified material were assessed in each OCT frame of MV and SB pullback by an imaging expert (YSC). Of note, the imaging expert was blind to the simulation results of the testing group. Then, the MV and SB were divided into sequential zones of heterogeneous plaque material and assigned with a quarter number (e.g. − 0.25, 0, + 0.25 etc.) ranging from + 2 (calcium only, very stiff) to − 2 (lipid only, very soft)^18^. The normal wall thickness and tapering were assessed by OCT. The normal wall material was modeled using the sixth-order reduced polynomial constitutive equation to characterize the isotropic hyper-elastic mechanical behavior, as previously described^20^. The material coefficients for normal arterial wall and plaque are listed in Table 2. The nickel cobalt alloy MP35N of Resolute Integrity and Onyx was modeled with the Von Mises-Hill plasticity model with isotropic hardening, while the Pt–Ir alloy core of Resolute Onyx was modeled with perfect plasticity. The material coefficients for the two alloys are listed in Table 2. The balloons were modeled as pure linear elastic materials with the same material properties as in simulations of the bench group.

*Stent and balloon crimping, positioning, and bending* All stents were first crimped from their nominal states using surface elements driven by radial displacement^18^. The crimped stents and balloons were positioned and bent along the centerline (Fig. 1**)**. The stent and balloons were precisely positioned within the bifurcations with reference to fiduciary markers (i.e. radiopaque markers of stent/balloons, carina, and intersection points of guidewires) in angiography and OCT. Since the information on the exact location of side branch recrossing was not available, in the computational stenting simulations, we recrossed the side branch through distal struts.

*Computational simulations* All steps of the PCI procedures were computationally replicated through a multi-step, large-deformation, quasi-static finite element analysis using the central difference method (Abaqus/Explicit solver; Table 3)^18^. In all analyses, the duration of balloon inflation and deflation was set to 0.05 s and the target time increments were set as 5 × 10^–8^ s (adjusted via mass scaling) to obtain fast quasi-static results while avoiding dynamic effects. The boundary conditions, simulation parameters and computer cluster described above in “Computational simulation of bench bifurcation stenting” section was used to perform the computational simulations.

#### 3D stent reconstruction from OCT

The stents were 3D reconstructed from OCT and angiographic images using a custom-built Grasshopper Python code^21^. First, the stent struts were segmented as individual points and flattened to 2D surfaces. Using the 2D stent design pattern as reference, the stent points were connected by lines that represented the centerlines of stent struts and links. Then, the 2D stent centerlines were wrapped and mapped back to the 3D lumen centerline, and finally, the volume of stent struts was added.

#### Computational fluid dynamic studies

The post-computational PCI bifurcation and stent geometries were used to discretize the fluid domain for CFD analyses (Fig. 1)^21–23^. The fluid domain was meshed with tetrahedral elements using ICEM CFD (ANSYS Inc.). Transient CFD simulations were performed with Fluent (ANSYS Inc.). Pulsatile flow was applied at the inlet of each artery^24^, and the inlet velocity was adjusted according to the inlet diameter^25^. To minimize the effect of boundary conditions, we added extensions (length = 10 times of the inlet diameter) to the inlet and outlet sections. The Huo-Kassab (HK) law^26^ was used to derive the relation between the diameter ratio of two daughter branches and the flow ratio through the branches. The lumen and stent surfaces were approximated as a rigid body, where non-slip boundary conditions were applied. The blood density was considered constant with a value of 1060 kg/m^3^^22^. The Carreau model was adopted to consider the non-Newtonian nature of blood. The following values for each parameter were used^27^: ${\mu }_{\infty }=0.0035\,{\text{Pa}}\,{\text{s}}$, ${\mu }_{0}=0.25\,{\text{Pa}}\,{\text{s}}$, $\lambda =25\,{\text{s}}$ and $n=0.25$. Three full cardiac cycles were simulated, and the results of the last cycle were used^28^.

#### Comparison metrics

The final lumen and stent geometries after computational stenting simulations were compared to the lumen and stent cross-sections segmented on post-PCI OCT. The cross-sections from post-PCI OCT were used as reference. The simulated bifurcation and frame number of OCT cross-sections were co-registered using carina as the fixed marker. The MLD along the stented MV and the mean stent diameter (MSD) were used as comparison metrics.

### Statistical methods

Statistical analysis was performed with the statistical package GraphPad Prism 8.0 (GraphPad Inc., San Diego, CA, USA). Continuous variables were expressed as mean ± standard error of mean. Bland–Altman analysis was used to measure agreement for method comparison studies.

## Results

### Bench simulations

**Figure 2:**
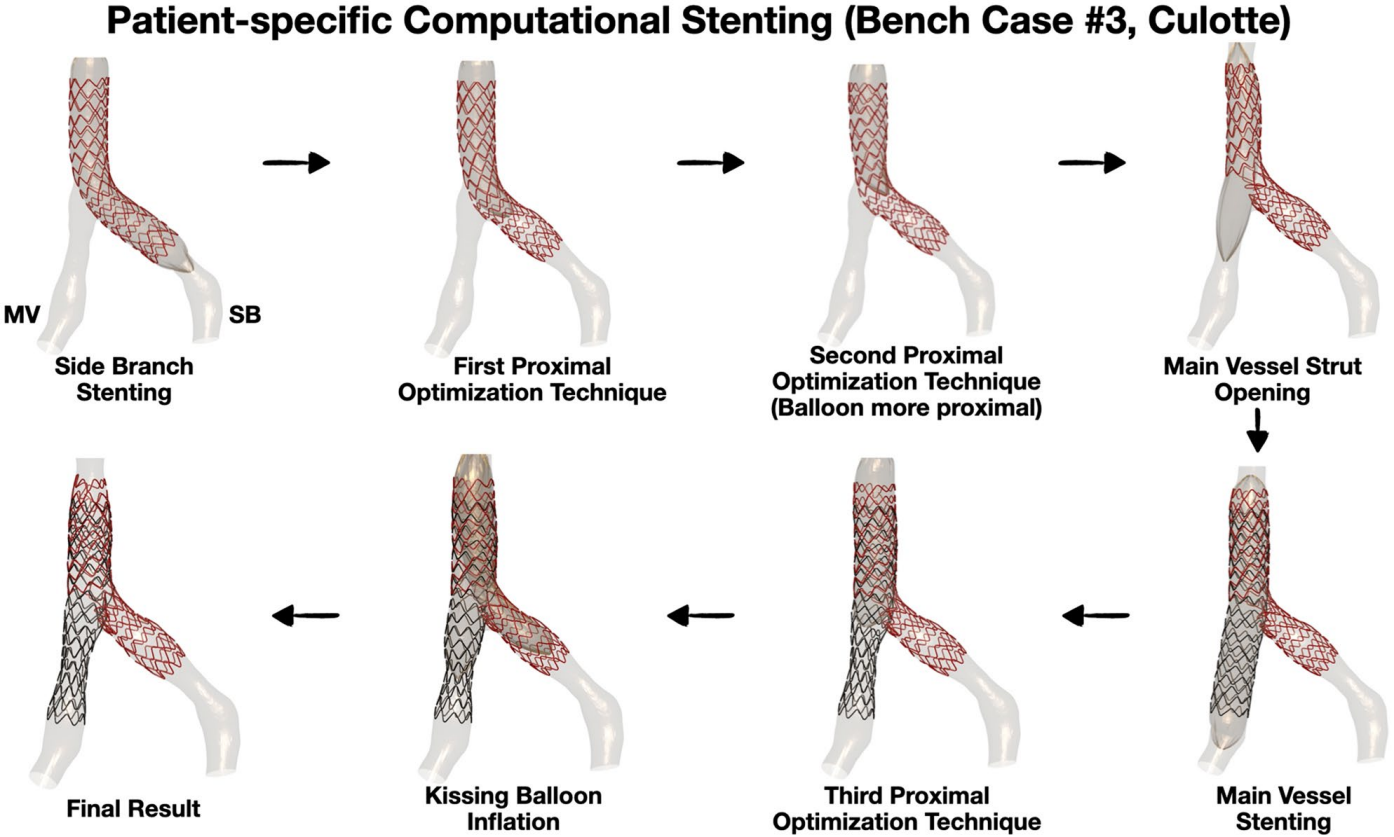
Representative computational simulation of a bench stenting case. A patient-specific silicone bifurcation geometry was stented on the bench with the culotte technique. We faithfully replicated computationally all the steps of the stenting procedure using the same bifurcation geometry and stent/balloon designs; *SB* side branch, *MV* main vessel.

**Figure 3:**
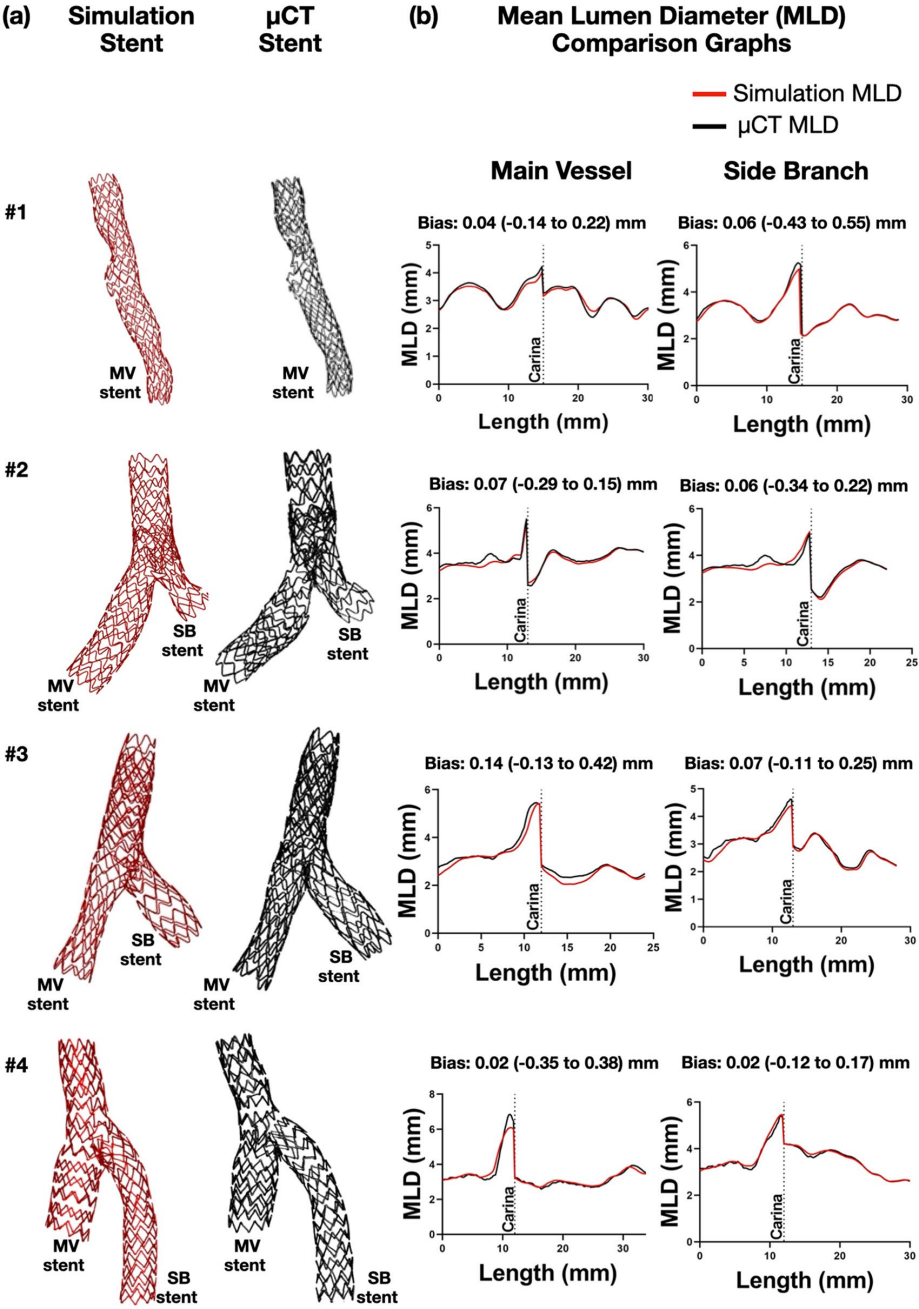
Computational stenting simulation versus µCT (Bench cases). Qualitative (**a**) and morphometric (**b**) comparison of computational stenting simulations against bench stenting imaged by μCT. Graphs show the stented part of the lumen only. Bias refers to the average difference in mean lumen diameters (MLD; 95% limits of agreement) between methods by Bland–Altman analysis; *MV* main vessel, *SB* side branch, *µCT* micro-computed tomography.

All bench stenting procedures, the majority of which were multi-step two- stent techniques, were successfully simulated. Figure 2 illustrates a representative example of Culotte technique with two stents. Visually, the computationally simulated stents were nearly identical in size and shape to the actual μCT-reconstructed stents (Fig. 3a). The MLD was plotted along the axial direction of the simulated and μCT reconstructed stents (Fig. 3b), and quantitatively compared the agreement between methods with Bland–Altman analysis that yielded an overall mean difference of 0.03 (− 0.28 to 0.34) mm (Table 4).

**Table 4:** Comparison of mean lumen diameter between computational stenting versus micro-computed tomography in bench cases by Bland–Altman analysis.

**Mean lumen diameter**		
Cases	Bias (mm)	95% limits of agreement (mm)
Bench #1		
MV	0.04	− 0.14 to 0.22
SB	0.06	− 0.43 to 0.55
Bench #2		
MV	0.07	− 0.29 to 0.15
SB	0.06	− 0.34 to 0.22
Bench #3		
MV	0.14	− 0.13 to 0.42
SB	0.07	− 0.11 to 0.25
Bench #4		
MV	0.02	− 0.35 to 0.38
SB	0.02	− 0.12 to 0.17
**Overall bias**	**0.03**	− **0.28 to 0.34**

**Figure 4:**
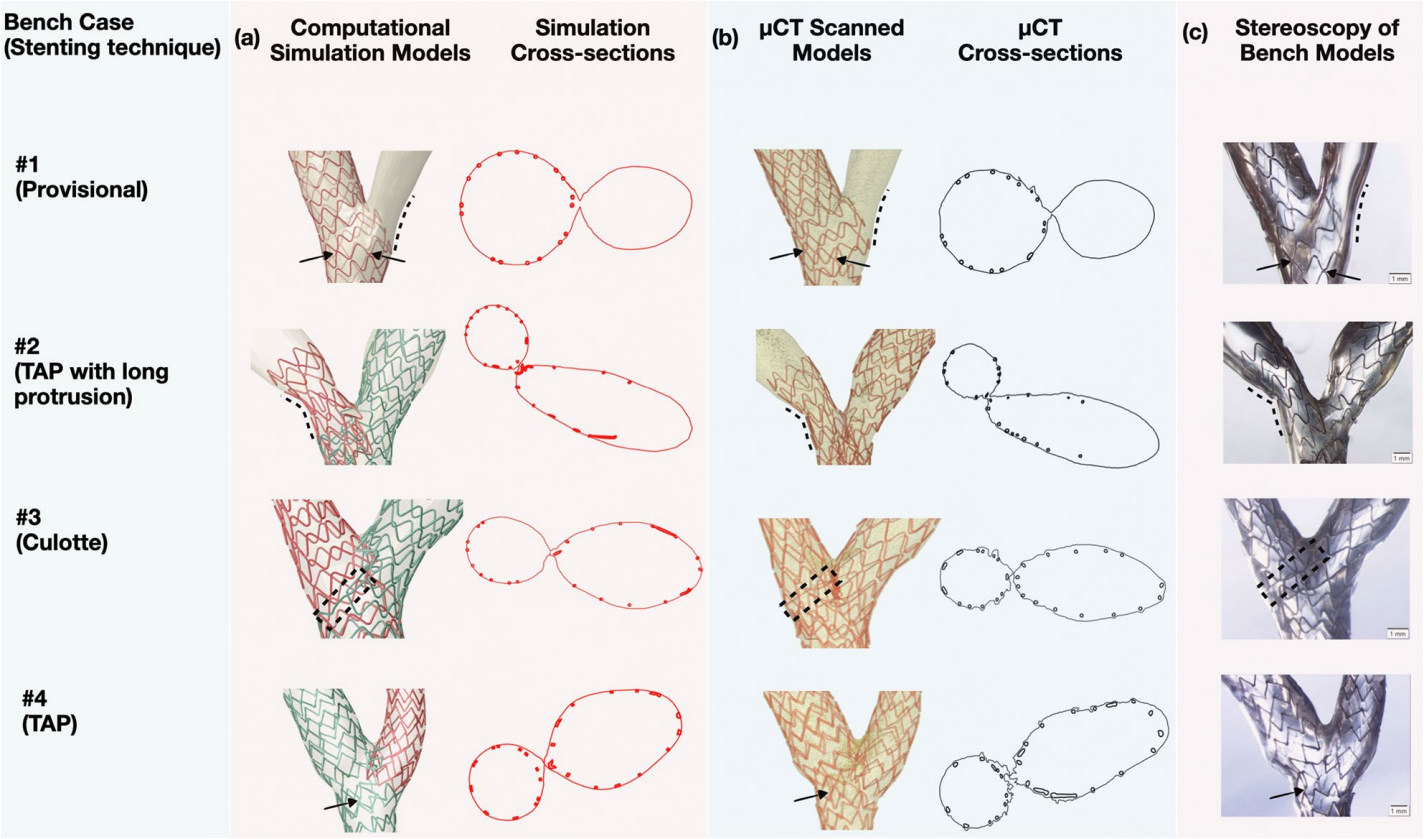
Qualitative comparison of computational stenting simulation of bench bifurcation models at the carina. Computational stenting (**a**) compared with bench stenting imaged by μCT (**b**) and stereoscopy (**c**) at the anatomically sensitive site of the carina. Simulation cross-sections (red) compared with the cross-sections from the µCT reconstructed model (black) showing similarity in the lumen shape at carina. In case #1 note the similarity of two stent links (black arrows), and the vessel curvature (black dotted line) in simulation and µCT and stereoscopic image. In case #2, note the similarity in vessel curvature (black dotted line). In case #3, note the very similar link position and shape (black dotted rectangle) at the bifurcation. In case #4, note the similarity in the position of stent link (black arrow); *TAP* T and protrusion, *µCT* micro-computed tomography.

Contrast-enhanced μCT and stereoscopic images further revealed the ability of our computational stenting platform to replicate, with high precision, fine details of the bench stenting procedures, including malapposed struts, side branch ostium size and shape, and gaps in struts around the anatomically sensitive site of the carina (Fig. 4).

### Clinical simulations

#### Training group

In the training group, the clinical stenting procedures were successfully simulated with our computational platform. The one-stent technique was followed for all of these procedures. Online Figure 3 summarizes all the steps of a representative case (Patient #1), in which provisional stenting technique with POT was followed. The Online Video shows the procedural steps of the case of patient #5. Visually, the computationally stented bifurcation lumen yielded high qualitative agreement with the angiographic lumen post stenting (Online Figure 4). Bland–Altman analysis revealed MLD differences close to zero [mean bias 0.08 mm (− 0.24 to 0.41) mm; Table 5, Online Figure 5]. Similarly, the computationally simulated stents exhibited high similarity to the shape and size of the actual stents which were 3D reconstructed by fusing OCT and angiography (Online Figures 6a and 6b). The MSD of the computationally simulated stents was quantitatively compared to the OCT stent segmentations, yielding very high agreement [mean bias 0.13 mm (− 0.21 to 0.48) mm; Table 6, Online Figures 5 and 6c]. Notably, in Patient #1, the computational simulation replicated the stent under-expansion around the carina secondary to the local stiff plaque material. In Patient #5, the computational stenting reproduced the large gaps between stent struts and consequent over-dilated lumen at the proximal MV following the proximal stent post-dilatation.

**Table 5:** Comparison of mean lumen diameter between computational stenting versus optical coherence tomography in clinical cases by Bland–Altman analysis.

**Mean lumen diameter**		
Training group	Bias (mm)	95% limits of agreement (mm)
Patient #1	0.10	− 0.27 to 0.47
Patient #2	0.05	− 0.12 to 0.23
Patient #3	0.07	− 0.34 to 0.48
Patient #4	0.03	− 0.22 to 0.28
Patient #5	0.20	0.05 to 0.34
** Overall bias**	**0.08**	− **0.24 to 0.41**
**Testing group**		
Patient #6	0.13	− 0.19 to 0.44
Patient #7	0.11	− 0.21 to 0.42
Patient #8	0.03	− 0.25 to 0.30
Patient #9	0.18	− 0.30 to 0.66
Patient #10	0.01	− 0.31 to 0.33
** Overall bias**	**0.08**	− **0.29 to 0.46**

**Table 6:** Comparison of mean stent diameter between computational stenting versus optical coherence tomography in clinical cases by Bland–Altman analysis.

**Mean stent diameter**		
Training group	Bias (mm)	95% limits of agreement (mm)
Patient #1	0.14	− 0.26 to 0.55
Patient #2	0.11	− 0.16 to 0.38
Patient #3	0.13	− 0.25 to 0.52
Patient #4	0.09	− 0.25 to 0.44
Patient #5	0.19	− 0.0004 to 0.39
** Overall bias**	**0.13**	− **0.21 to 0.48**
**Testing group**		
Patient #6	0.19	− 0.11 to 0.48
Patient #7	0.16	− 0.14 to 0.46
Patient #8	0.03	− 0.37 to 0.43
Patient #9	0.23	− 0.28 to 0.74
Patient #10	0.09	− 0.25 to 0.43
** Overall bias**	**0.14**	− **0.25 to 0.54**

#### Testing group

**Figure 5:**
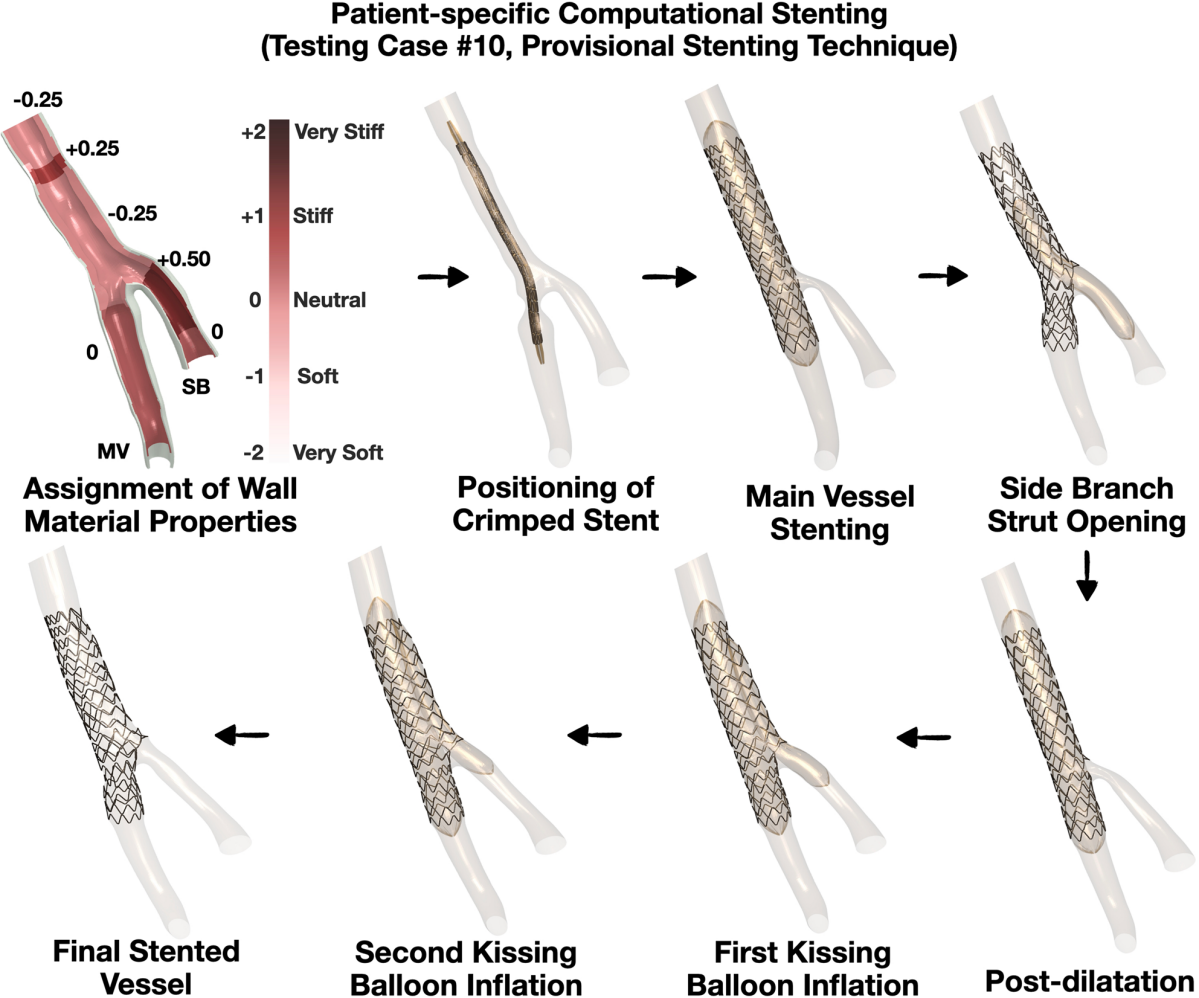
Representative computational stent simulation of a clinical case (Testing group). A patient-specific bifurcation geometry was stented clinically following the provisional technique. We faithfully replicated computationally all the steps of the stenting procedure using the same bifurcation anatomy, wall material properties, stent/balloon designs, inflation pressure and stenting technique. Note that the computational simulation was blinded to the post-procedural OCT. Material properties were assigned to the 3D reconstructed bifurcation wall based on OCT imaging (Vessel in top left). Wall stiffness score varied from − 0.25 (Fibrolipid material) to + 0.50 (Fibrocalcific material); *MV* main vessel, *SB* side branch.

**Figure 6:**
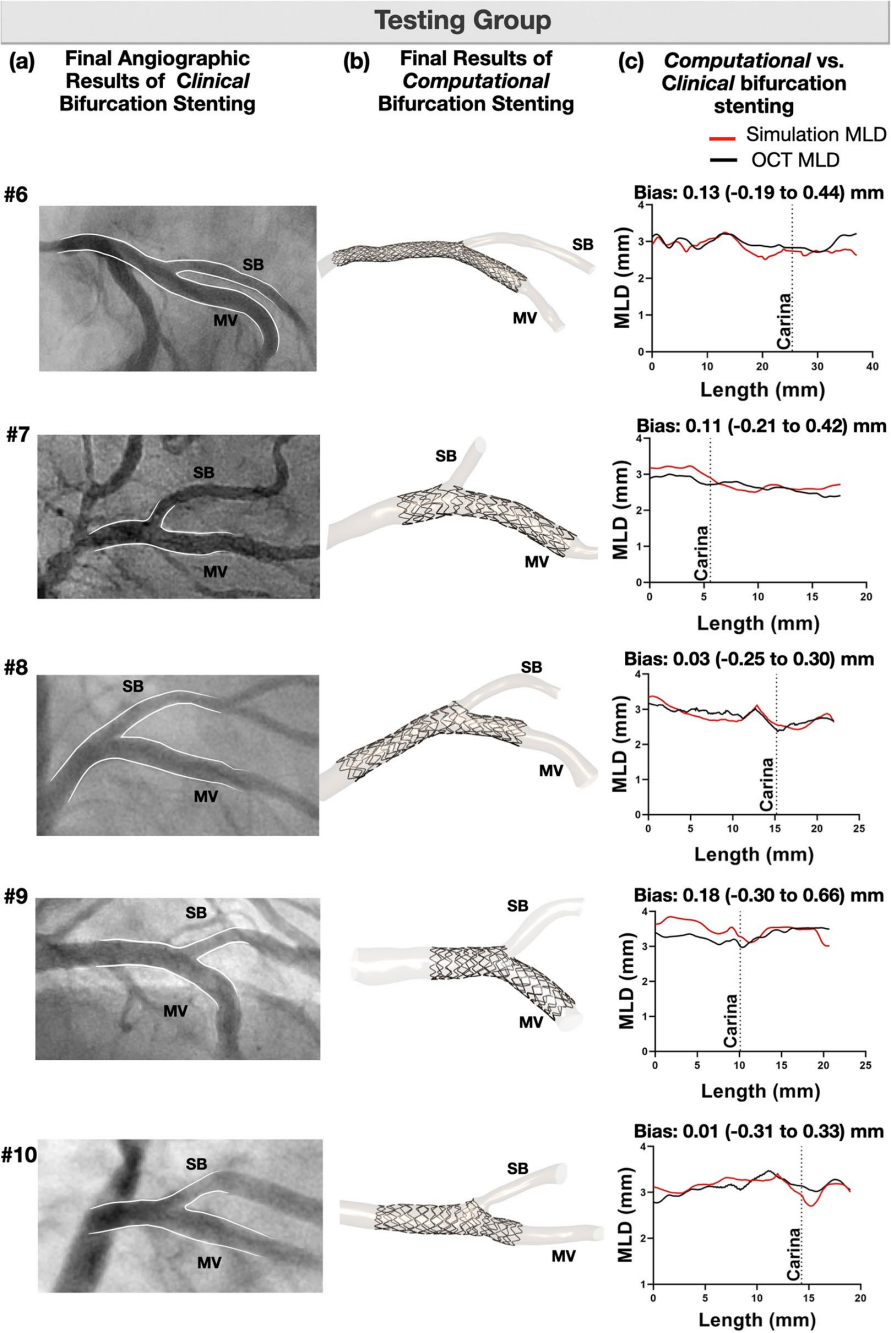
Blind comparison of computational stenting simulation versus angiography and OCT (Testing group). Qualitative (**a**, **b**), and quantitative (**c**) comparison of lumen size after computational stenting against angiography and OCT imaging. The graphs include the stented part of the lumen. Note that the computational stent simulation was blind to the final OCT results post-procedure; *MV* main vessel, *SB* side branch, *MLD* mean lumen diameter.

**Figure 7:**
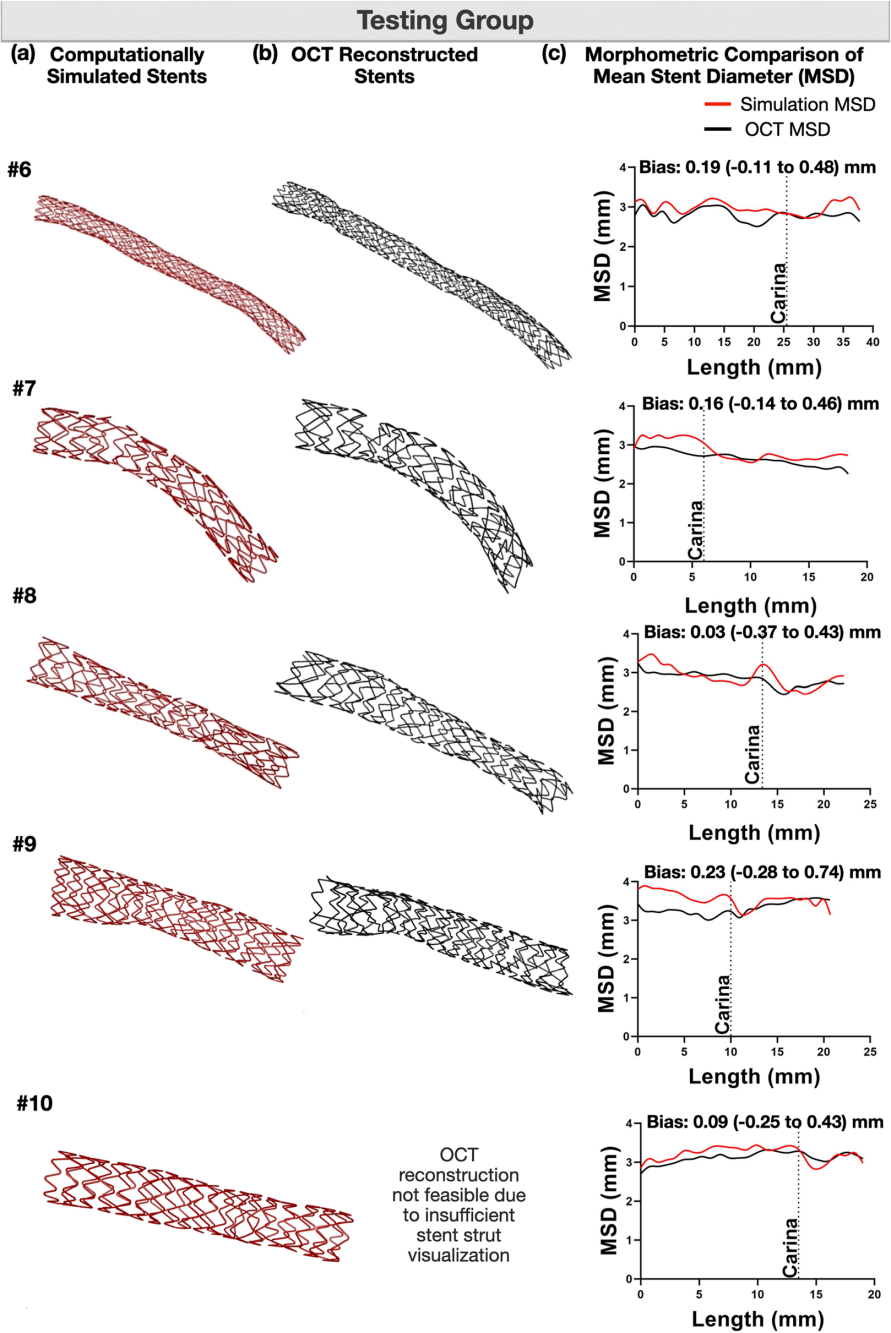
Blind computational stenting simulation versus OCT (Testing group). Qualitative (**a**, **b**), and quantitative (**c**) comparison of mean stent diameter (MSD) after computational stenting against OCT. The stents in (**b**) were 3D reconstructed from OCT^21^. Note that the computational stent simulation was blind to the final OCT results post-procedure.

**Figure 8:**
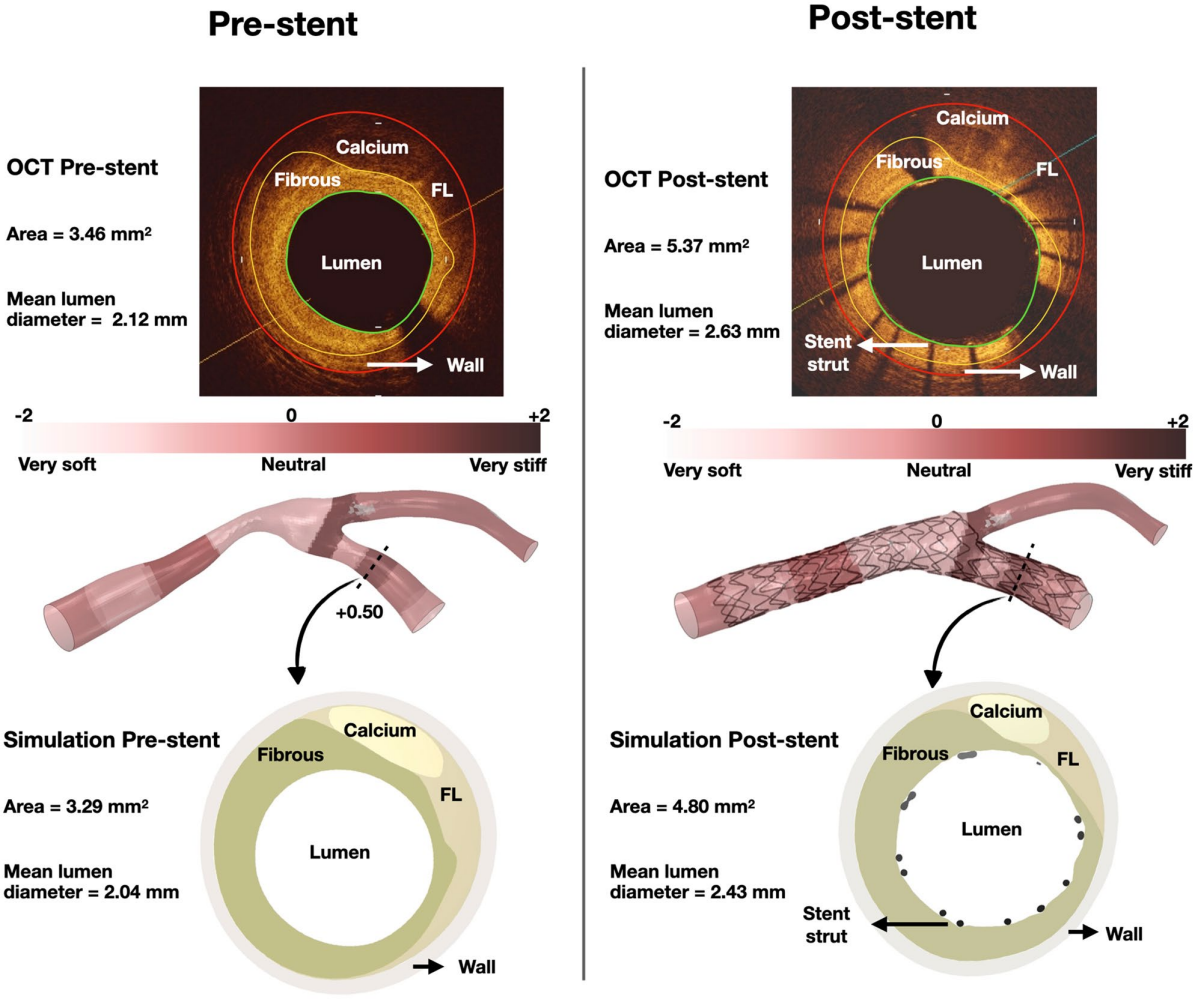
Representative example showing the similarity between computational stenting simulation and reality (OCT). Left column shows the lumen size pre-stenting and right column shows the lumen size post-stenting. The figure illustrates the pre-procedural 3D reconstructed vessel anatomy and plaque stiffness, as well as the post-procedural stented vessel. Note the similarity in pre-stenting lumen size, shape and plaque constituents by OCT versus computational simulation. In the computational simulation, this vessel cross-section was assigned + 0.50 plaque stiffness based on OCT imaging. Likewise, note the similarity in post-stenting lumen size and shape, as well as stent size and circumferential configuration by OCT and computational simulation; *FL* fibrolipid.

In the testing group, we used only the pre-procedural anatomical information (angiography and OCT) to assess the ability of our computational platform to replicate the clinical stenting **(**Fig. 5**)**. The computational simulation operators were blinded to the post-procedural OCT. As shown in Figs. 6 and 7, computational stenting yielded very high agreement to post-procedural OCT suggesting the robustness of our platform. Quantitative comparisons by Bland–Altman analysis showed small differences in MLD and MSD [mean bias 0.08 mm (− 0.29 to 0.46) mm and 0.14 mm (− 0.25 to 0.54) mm, respectively; Tables 5 and 6, Online Figure 5]. Figure 8 shows a representative example of a blinded testing case. Pre-procedural OCT provided anatomical inputs (lumen area, plaque thickness, plaque eccentricity and plaque material properties) in our computational platform. Patient-specific computational stent simulations yielded similar lumen and stent expansion with the post-procedural OCT.

#### Processing times

The average processing times from model preparation  to completion of the computational simulations are provided in Table 7.

**Table 7:** Average processing times for computational stenting simulations for clinical cases.

	Hours
**Model preparation**	
1. Crimping of stent and artery meshing	2.0 ± 0.25
2. Material properties assignment and parameters setting	1.0 ± 0.25
**Computational simulation**	
1. Locating stent and balloon	0.5 ± 0.25
2. Bending of stent and balloon	0.5 ± 0.25
3. Running the simulation of the numerical model	6.0 ± 1.00
**Total time for computational simulation of each step**	**7.0** ± **1.50**

#### CFD studies

To show the feasibility of CFD in our simulated procedures, we compared the time-averaged wall shear stress (TAWSS) along the axial direction of MV and SB before and after computational stenting (Online Figure 7). As shown quantitatively and qualitatively, stenting smoothed the wall shear stress pattern along the MV, resulting in lower time averaged wall shear stress at the stenosis.

## Discussion

In this work, we presented and validated a novel, fully computational methodology for patient-specific stenting simulations of coronary artery bifurcations. Our computational simulation platform was trained using patient-specific bench and clinical cases, covering a wide spectrum of bifurcation disease complexity, stenting techniques (1- and 2-stent techniques), and drug-eluting stent platforms (zotarolimus- and everolimus-eluting stents). The accuracy of our platform was tested blindly against clinical cases. High resolution imaging of bench and clinical cases with μCT and OCT, respectively, was used as the ground truth for the training and testing of computational stenting. Collectively, our studies demonstrate that computational bifurcation stenting is feasible and accurate. To the best of our knowledge, this study is the first to present and validate a full pipeline from bifurcation imaging to computational 3D reconstruction, computational stent deployment (using realistic plaque properties), and CFD analysis. Furthermore, this is the first study in which multiple stenting techniques and stent platforms were simulated in patient-specific bifurcation geometries. Previous attempts to virtually implant stents in patient-specific bifurcation models had several limitations (simplified 1-stent technique, inaccurate representation of true bifurcation anatomy and plaque material properties, and lack of blinded validation)^11,20^.

Of note, the ability of our computational stenting platform to perform realistic stent simulations was exhibited in clinical cases, in which the operators of computational simulations were blinded to post-procedural imaging. The feasibility and robustness of our platform was based on two important features: (i) Precise anatomical representation of the bifurcation, and (ii) Assignment of realistic plaque anatomy and material properties. We used either μCT or OCT for the anatomical representation of bifurcation, paying attention to the accurate reconstruction of the anatomically sensitive region of carina. Other imaging modalities, including intravascular ultrasound or coronary computed tomography angiography could also perform well with our platform. Unlike previous studies, we employed a sophisticated approach to achieve realistic vessel response to stent and balloon expansion by integrating the following plaque morphology components: (i) Pre-procedural lumen stenosis, (ii) Plaque thickness, (iii) Plaque eccentricity, and (iv) Plaque tissue characteristics.

Another key feature of our computational stenting platform is the versatility across different stenting techniques and stent platforms/sizes. In the patient-specific bench models, our computational approach accurately replicated all the steps of almost all the guideline-proposed bifurcation stenting techniques^29^, i.e. provisional, T-and-protrusion and culotte followed by POT and KBI (detailed steps are shown in Table 1). In the clinical cases, we performed provisional stenting followed by KBI or POT (detailed steps are shown in Table 3). In all these computational simulations, different stent platforms were used, including the laser-cut everolimus-eluting stents, wire stent with circular strut cross-section (Resolute Integrity), and wire stent with shell-core strut cross-section (Resolute Onyx). Overall, our computational simulations (particularly the 2-stent techniques) involved multiple complicated contacts, i.e. balloon-stent, balloon-artery, stent-artery, stent-stent, balloon-balloon, and multiple steps with each step serving as input for the next one. These non-linear contacts could create computational and geometrical errors multiplied and propagated throughout the steps if they were not handled correctly. The incorporation of accurate bifurcation anatomy and plaque morphology were fundamental for the successful completion of our multi-step, quasi-static, finite element analyses and one of the major novelties in this work. Notably, we replicated the correct stent position not only in the longitudinal direction, but also in the circumferential direction. This is of paramount clinical importance when it comes to jailing of SB ostium **(**Fig. 4**)**.

### Clinical perspectives

The proposed computational platform for patient-specific bifurcation stenting simulations provides a reliable resource for clinical research, clinical decision making, stent manufacturing and education on stenting techniques. Computational stenting can be used in virtual (in-silico) clinical trials using patient-specific anatomical and physiological data and provide surrogate endpoints (i.e. under-expansion, malapposition, flow dynamics) that are highly predictive of clinical endpoints. These virtual clinical trials can be adequately powered with large volume patient data to investigate the performance of different stenting techniques or stent platforms, thereby guiding the actual clinical trials. Flow ISR study, which is currently underway, is an example of such a virtual clinical trial. The study compares different 1- and 2-stent techniques in patient-specific coronary bifurcations. In cardiac catheterization laboratory, computational stenting simulations can be used for pre-procedural planning and decision-making. Computational simulation assisted identification of the optimal stent strategy can provide invaluable guidance to the interventionalist to increase the procedural success and achieve favorable long-term clinical outcomes. When it comes to stent manufacturers, a cost- and time-effective computational stenting strategy using patient-specific anatomies has the potential to reduce the need for bench and animal research for stent testing. Computational simulations can help with stent design optimization (e.g. number of crowns and links, strut size) and mechanics (radial and longitudinal strength, expansion capability, vessel scaffolding)^18^. The computational approach can effectively evaluate different stent designs in realistic vessel environments obviating the need to manufacture and experimentally test stent prototypes, ultimately reducing the development time and manufacturing costs. Another important consideration with computational stenting is that it can be used as an educational tool to train healthcare providers on bifurcation stenting techniques. Extended reality technologies can further assist towards this direction. Finally, computational bifurcation stenting can be translated to other vascular beds (e.g. carotid, renal or aortic bifurcations) and structural heart interventions.

### Limitations

There are several limitations within our study. First, in simulations of clinical cases the vessel motion related to cardiac cycle was not considered. Adding the parameter of time (4D simulation) would potentially make the simulation more realistic, at the expense of increased computational time. The tradeoff between improved accuracy and increased computational time warrants further investigation. Second, in rare cases with very tortuous or complex coronary geometries that are not clearly visualized on two angiographic views (at least 30 degrees apart), 3D reconstruction of the bifurcation and consequently computational stenting simulation might be limited. Third, in the clinical cases, plaque was 3D reconstructed by OCT. We acknowledge that OCT is not the best modality to visualize the outer vessel; however, in the majority of OCT frames we were able to identify the outer plaque borders with confidence. Fourth, the processing time was about 7.0 ± 1.5 h per simulation step (Table 7). One of our priorities is to reduce the processing time by streamlining our computational methods and increasing the computer power and integrating machine learning techniques. Fifth, we made some assumptions in the simulations of bench cases: To avoid heavy computational load in the bench simulations, the silicone wall was meshed with shell elements. Since the silicone wall thickness varied in each model, the assigned shell thickness could deviate slightly from the real silicone wall thickness. However, no significant errors were expected from this assumption. The material properties of silicone were obtained by fitting the stress–strain curve from compression tests, which may differ than that derived from the tensile tests. Sixth, there were some technical assumptions in the simulations of clinical cases. The material properties of different plaque types were adopted from literature^19^. However, the literature data are quite representative of the entire spectrum of plaque material properties and we feel that they did not deviate from the true plaque properties. In accordance with previous studies^17,30^, we used the idealized multi-wing crimped balloon model. We adopted the material properties for compliant, semi-compliant and non-compliant balloons from literature^15^. The computational model could be improved if the material properties of the balloon were calibrated to the corresponding compliance data from the manufacturer^31^. The pre-stressed state of the arterial wall due to blood pressure was neglected. Finally, the primary purpose of this work was to virtually replicate the actual stenting steps. The reverse process of executing the computational simulation steps in the cardiac catheterization laboratory is a fascinating perspective that warrants to be tested in future work.

## Conclusions

Patient-specific computational stenting of coronary artery bifurcations is a feasible and accurate approach. Future studies are warranted to investigate the ability of computational stenting simulations to guide decision making in the cardiac catheterization laboratory and improve clinical outcomes.

## Supplementary Information

Supplementary Information.

Supplementary Information.

## Abbreviations

2D: Two-dimensional
3D: Three-dimensional
CFD: Computational fluid dynamics
IQR: Inter quartile range
KBI: Kissing balloon inflation
MLA: Mean lumen area
MLD: Mean lumen diameter
MSD: Mean stent diameter
MV: Main vessel
NC: Non-compliant
OCT: Optical coherence tomography
PCI: Percutaneous coronary intervention
POT: Proximal optimization technique
SB: Side branch
SC: Semi-compliant
TAWSS: Time-averaged wall shear stress
μCT: Micro-computed tomography
