# Supplementary material for: Patient-specific computational simulation of coronary artery bifurcation stenting
Source: Sci Rep. 2021 Aug 13;11:16486. doi: 10.1038/s41598-021-95026-2 (PMC8363606; doi:10.1038/s41598-021-95026-2)
Supplement: Supplementary file 1 — Supplementary Information. [file 41598_2021_95026_MOESM1_ESM.docx]

**Patient-Specific Computational Simulations of Coronary Artery Bifurcation Stenting**

Shijia Zhao, PhD^1*^; Wei Wu, PhD^1*^; Saurabhi Samant, MBBS^1^; Behram Khan, MD^1^; Ghassan S Kassab, PhD^2^; Yusuke Watanabe, MD^3^; Yoshinobu Murasato, MD, PhD^4^; Mohammadali Sharzehee, PhD^1^; Janaki Makadia, MBBS^1^; Daniel Zolty, BSc^1^; Anastasios Panagopoulos, MD^1^; Francesco Burzotta, MD^5^; Francesco Migliavacca, PhD^6^, Thomas W. Johnson, MD^7^; Thierry Lefevre, MD^8^; Jens Flensted Lassen, MD, PhD^9^; Emmanouil S. Brilakis, MD, PhD^10^; Deepak L. Bhatt, MD, MPH^11^; George Dangas, MD, PhD^12^; Claudio Chiastra, PhD^13^; Goran Stankovic, MD^14^;Yves Louvard, MD^15^; Yiannis S. Chatzizisis MD, PhD^1^

^1^Cardiovasclar Biology and Biomechanics Laboratory, Cardiovascular Division, University of Nebraska Medical Center, Omaha, Nebraska, United States

^2^California Medical Innovation Institute, San Diego, California, United States

^3^Department of Cardiology, Teikyo University Hospital, Tokyo, Japan

^4^Department of Cardiology, Kyushu Medical Center, Fukuoka, Japan

^5^Department of Cardiovascular Sciences, Fondazione Policlinico Universitario A. Gemelli IRCCS Università Cattolica del Sacro Cuore, Rome, Italy

^6^Laboratory of Biological Structure Mechanics (LaBS), Department of Chemistry, Materials and Chemical Engineering “Giulio Natta,” Politecnico di Milano, Milan, Italy

^7^Department of Cardiology, Bristol Heart Institute, University Hospitals Bristol NHSFT & University of Bristol, Bristol, United Kingdom

^8^Ramsay Générale de Santé - Institut cardiovasculaire Paris Sud, Hopital Privé Jacques Cartier, Massy, France

^9^Department of Cardiology B, Odense Universitets hospital & University of Southern Denmark, Odense C, Denmark

^10^Minneapolis Heart Institute, Minneapolis, Minnesota, United States

^11^Brigham & Women's Hospital, Harvard Medical School, Boston, Massachusetts, United States

^12^The Zena and Michael A. Wiener Cardiovascular Institute, Mount Sinai Hospital, Icahn School of Medicine, New York City, New York, United States

^13^PoliTo^BIO^Med Lab, Department of Mechanical and Aerospace Engineering, Politecnico di Torino, Turin, Italy

^14^Department of Cardiology, Clinical Center of Serbia, Belgrade, Serbia

^15^Institut Cardiovasculaire Paris Sud, Massy, France

*The first two authors contributed equally

**Address for Correspondence:**

*Yiannis S. Chatzizisis, MD, PhD*

Cardiovascular Biology and Biomechanics Laboratory

Cardiovascular Division

University of Nebraska Medical Center

Omaha, Nebraska, USA

Tel: (402) 559-5156

Fax: (402) 559-0710

E-mail: [ychatzizisis@icloud.com](mailto:ychatzizisis@icloud.com)

**Online Figures**

**Online Figure 1.** Study design of the training and blind testing of computational bifurcation stenting simulations showing number of cases and simulations carried for training and testing of the computational stenting simulation platform.

**
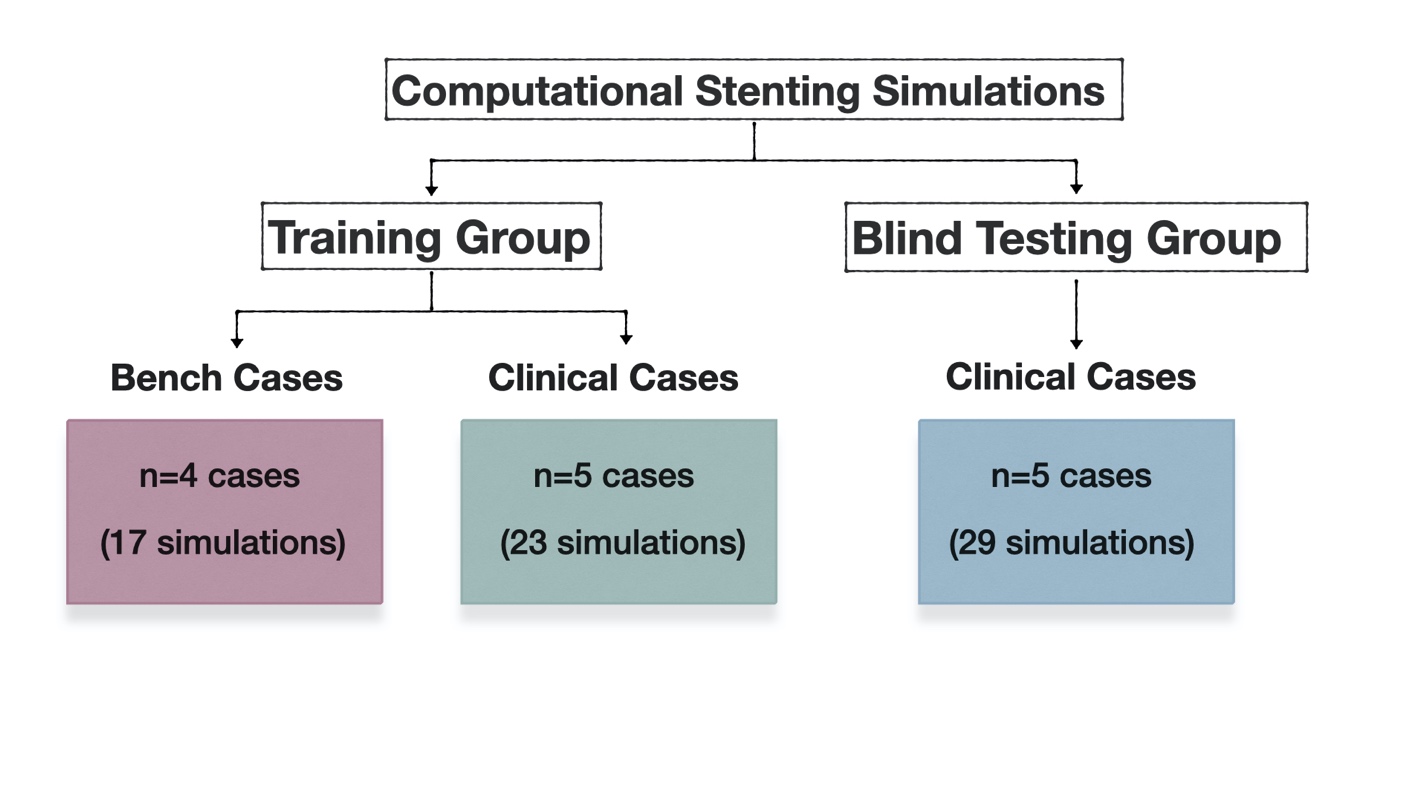
**

**Online Figure 2. Assignment of plaque material properties.** Based on the plaque constituents by OCT imaging we chose plaque material properties in our computational simulation platform. The plaque material properties were derived by fitting discrete functions to stress-strain curves obtained from experimental uniaxial tensile tests that were conducted with human cadaveric atherosclerotic tissues.

**
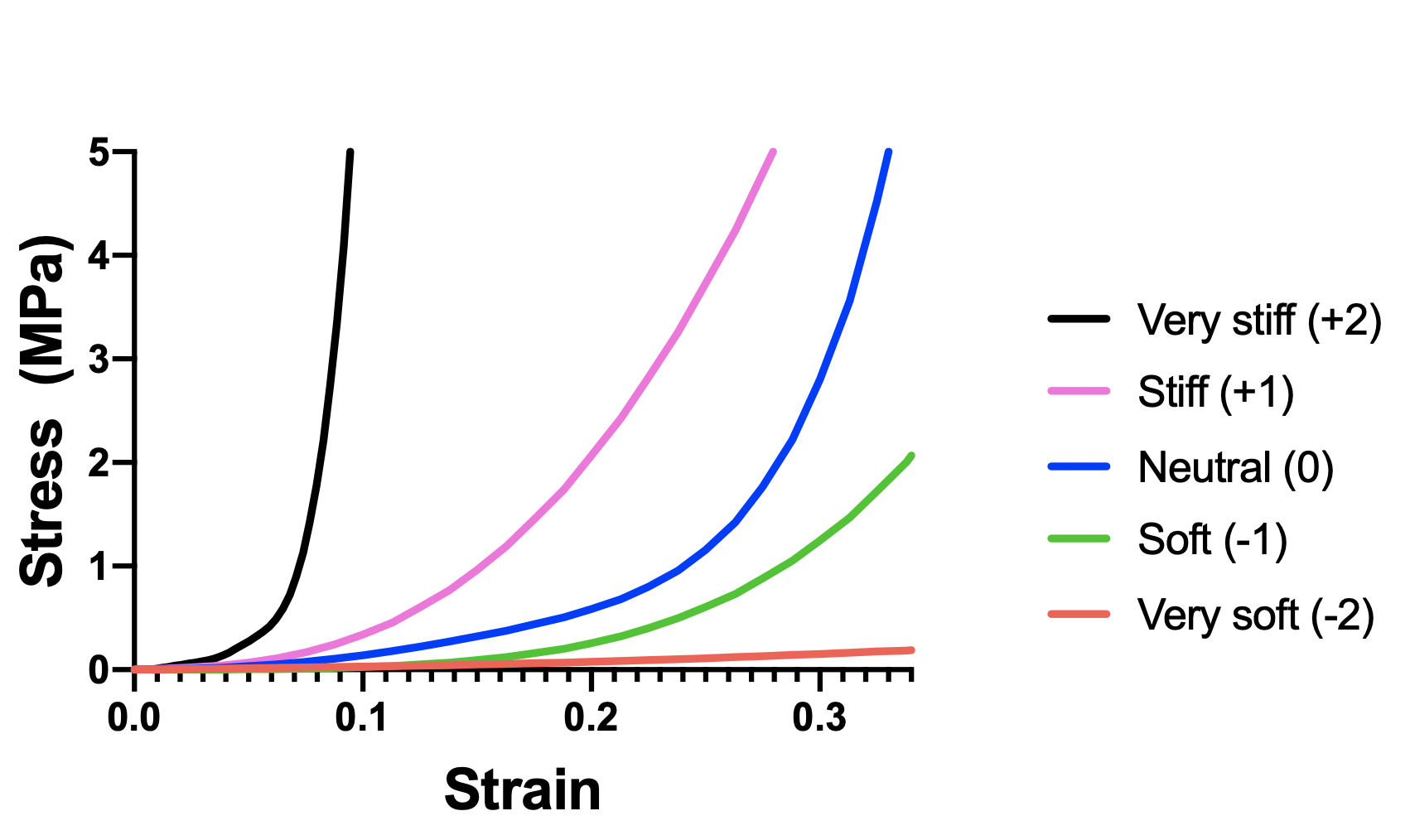
**

**Online Figure 3.** **Representative computational stent simulation of a clinical case (Training group).** A patient-specific bifurcation geometry was stented clinically with provisional technique. We faithfully replicated computationally all the steps of the stenting procedure using the same bifurcation anatomy, wall material properties, stent/balloon designs, inflation atmospheres and stenting technique. Material properties were assigned to the 3D reconstructed bifurcation wall based on OCT imaging; MV: main vessel, SB: side branch

**
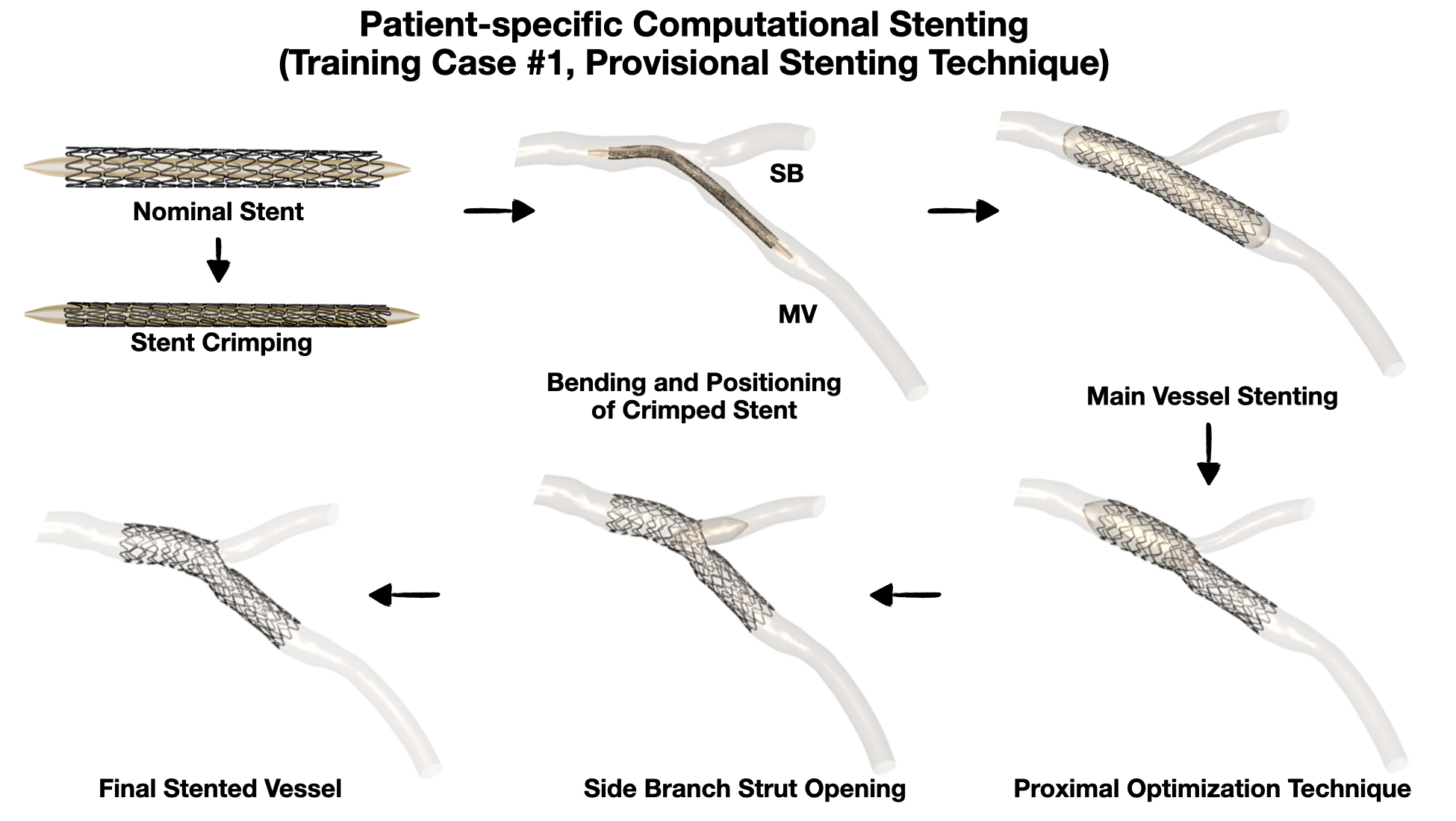
**

**Online Figure 4. Computational stenting simulation vs. angiography and OCT (Training group).** Qualitative (a & b), and quantitative (c) comparison of lumen size after computational stenting against angiography and OCT imaging. The graphs show the stented part of the lumen only; MLD: mean lumen diameter, MV: main vessel, SB: side branch.


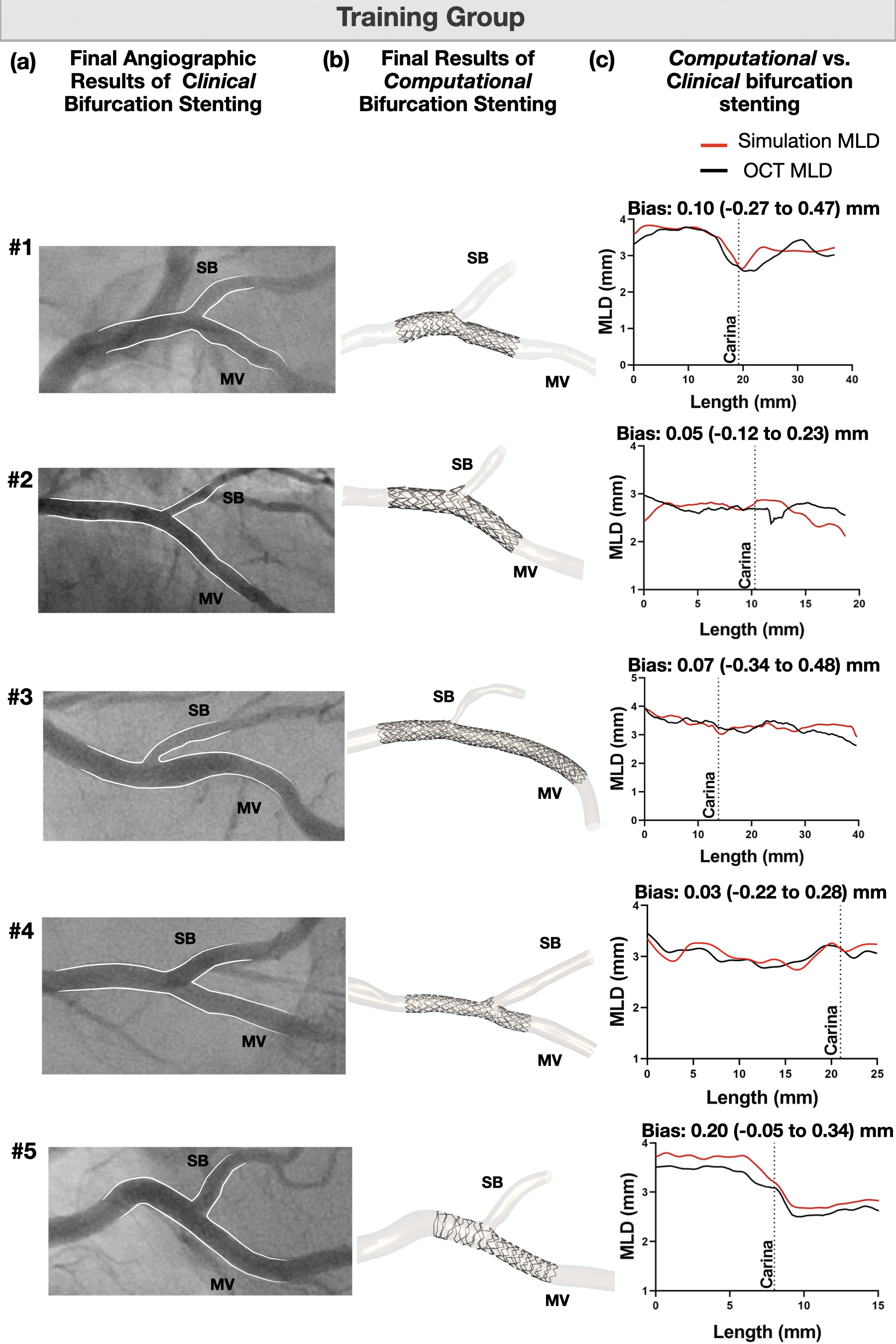


**Online Figure 5. Computational vs. clinical stenting.** Bland Altman analysis of computational vs. clinical stenting in training group (a) and blind testing group (b) yielding an overall very high agreement with biases at 0.08 mm for lumen size and <0.15 mm for stent size; MLD: mean lumen diameter, MSD: mean stent diameter


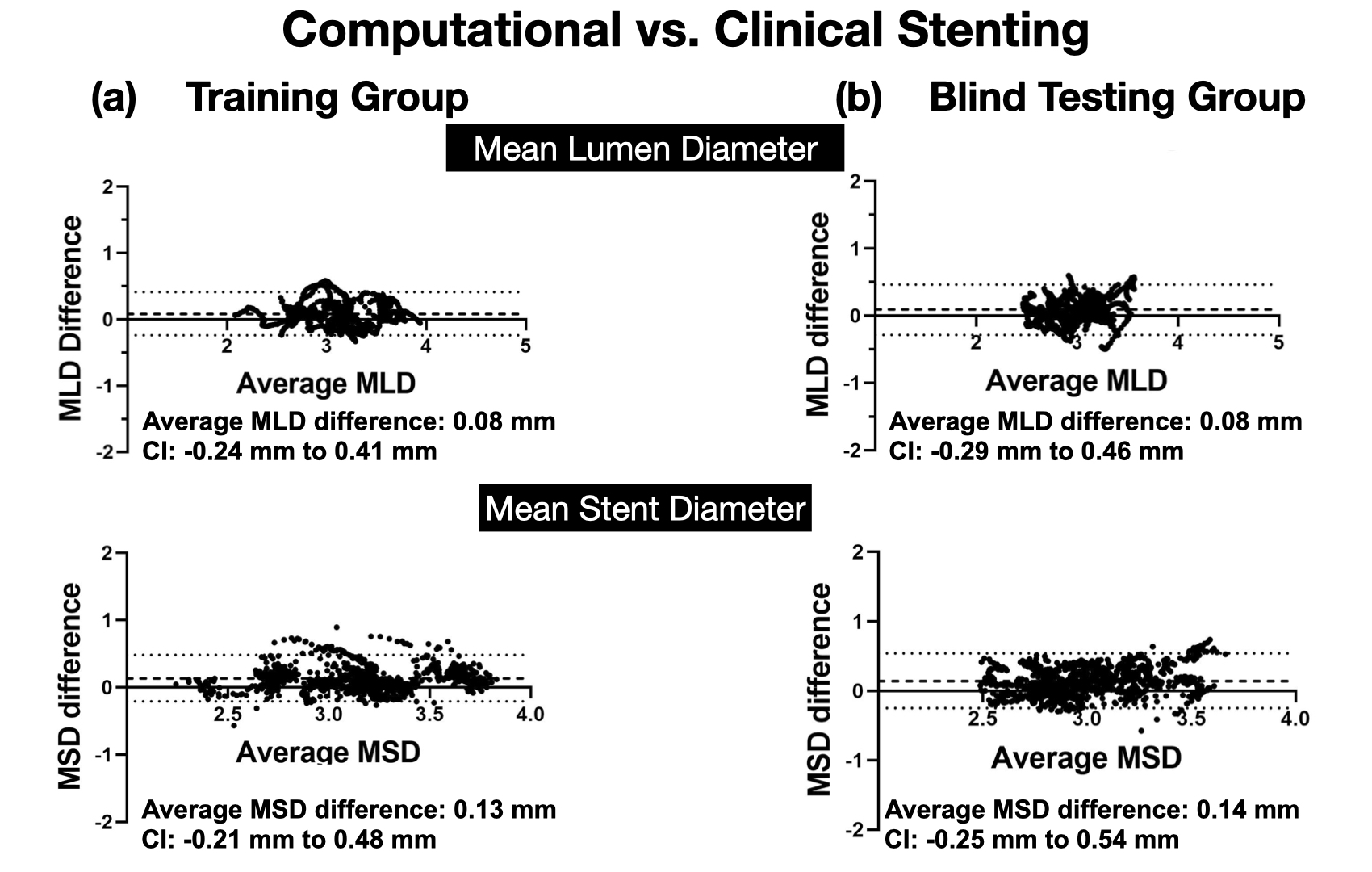


**Online Figure 6. Computational stenting simulation vs. OCT (Training group).** Qualitative (a and b), and quantitative (c) comparison of mean stent diameter (MSD) after computational stenting against OCT. Note that the stents in (b) are 3D reconstructed from OCT (1).


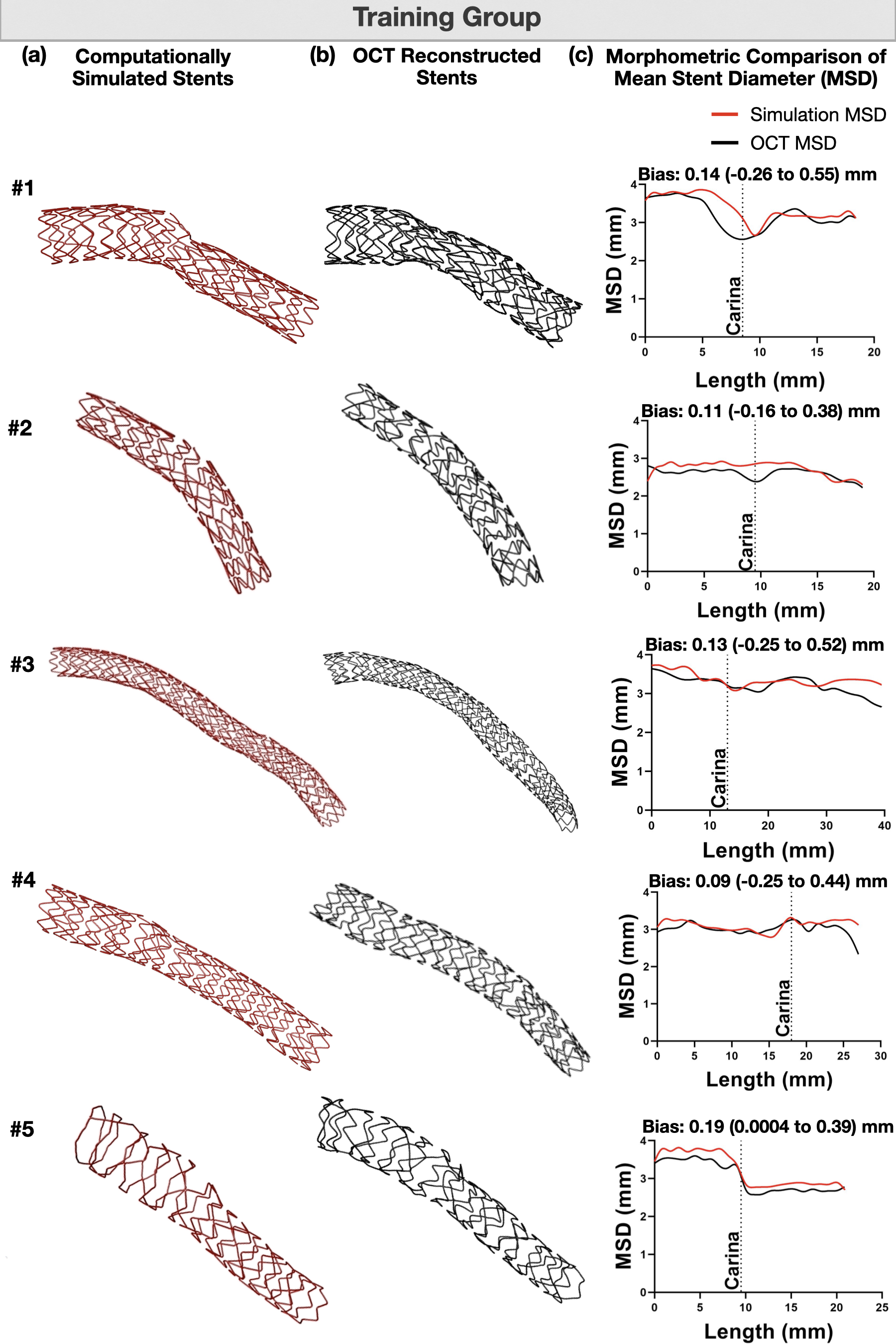


**Online Figure 7.** **Computational fluid dynamic studies pre- and post-computational stenting**. Qualitative (a) and quantitative (b) computational fluid dynamic studies comparing the time averaged wall shear stress before and after computational stenting. Graphs show the entire length of the reconstructed lumen; dashed lines indicate the carina and solid lines the stented part of the lumen. Note the restoration of more homogenous flow environment after stenting; MV: main vessel, SB: side branch, TAWSS: time-averaged wall shear stress


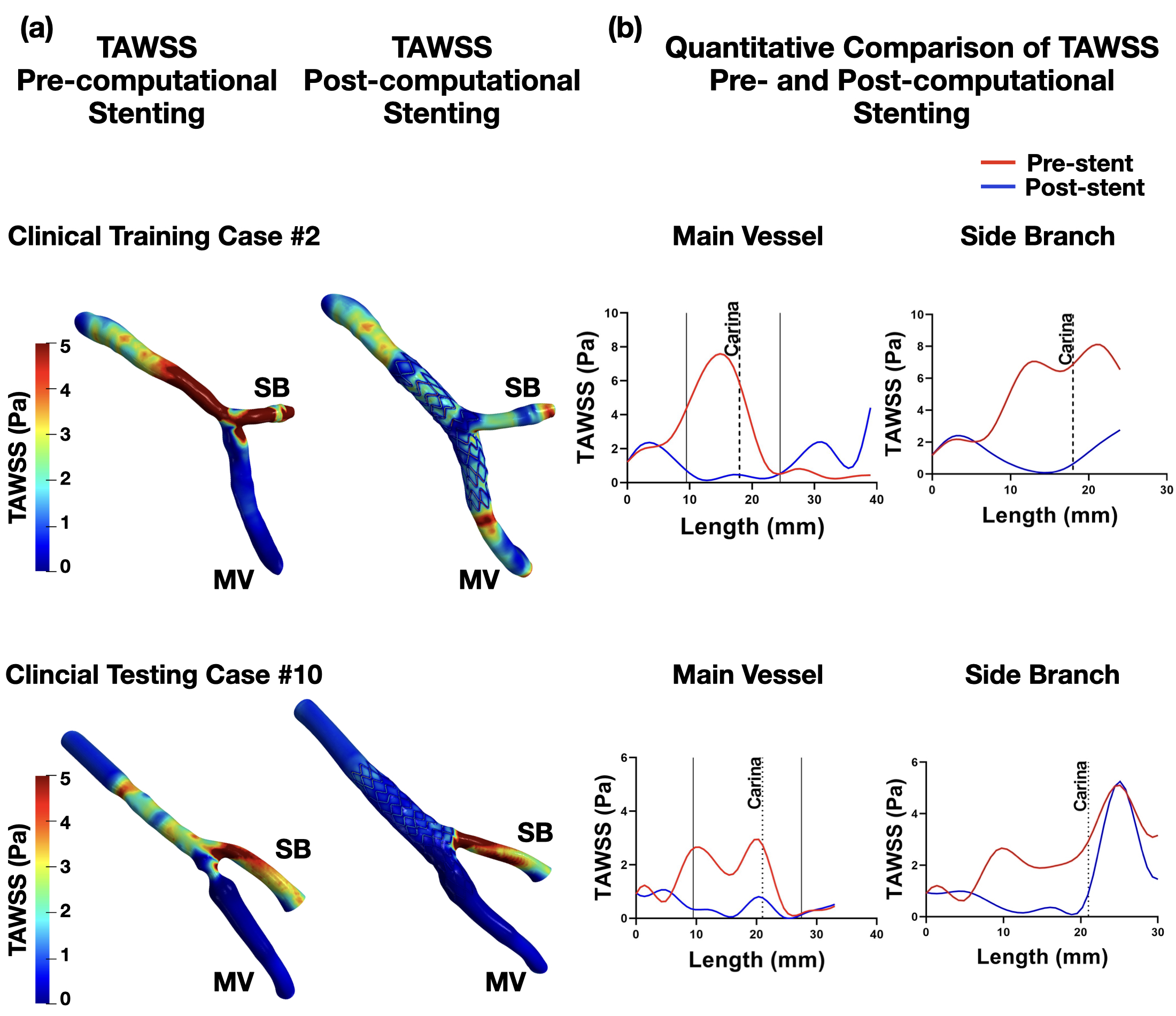


**Online Tables**

**Online Table 1.** Patient demographic and clinical characteristics of the testing and training cases; POT: proximal optimization technique, KBI: kissing balloon inflation

|  | Age  (years) | Gender | Cardiovascular comorbidities | Coronary bifurcation treated | | Stenting technique |
| --- | --- | --- | --- | --- | --- | --- |
| **Training cases** | | | | | | |
|  |  |  |  | **Main vessel** | **Side branch** |  |
| Patient #1 | 69 | Male | Hyperlipidemia | Left anterior descending artery | First diagonal branch | Provisional technique with POT |
| Patient #2 | 65 | Male | Hyperlipidemia, diabetes, and past history of smoking | Left anterior descending artery | First diagonal branch | Provisional technique with KBI |
| Patient #3 | 77 | Male | Hyperlipidemia, and past history of smoking | Left anterior descending artery | First diagonal branch | Provisional technique with POT |
| Patient #4 | 67 | Male | Hyperlipidemia, past history of heart failure and smoking | Right coronary artery | Posterior left branch | Provisional technique with POT + KBI |
| Patient #5 | 69 | Male | None | Left anterior descending artery | First diagonal branch | Provisional technique with POT |
| **Testing cases** | | | | | | |
| Patient #6 | 78 | Male | Hyperlipidemia, diabetes, | Left anterior descending artery | First diagonal branch | Provisional technique with POT + KBI |
| Patient #7 | 68 | Female | None | Left anterior descending artery | Second diagonal branch | Provisional technique with POT |
| Patient #8 | 69 | Female | Hyperlipidemia | Left anterior descending artery | First diagonal branch | Provisional technique with POT + KBI |
| Patient #9 | 49 | Male | Hyperlipidemia, diabetes, and past history of smoking | Left anterior descending artery | First diagonal branch | Provisional technique with POT |
| Patient #10 | 78 | Female | Diabetes | Left anterior descending artery | First diagonal branch | Provisional technique with KBI |

**Reference**

1. Wu W, Khan B, Sharzehee M et al. 3D Reconstruction of Coronary Artery Stents from Optical Coherence Tomography: Experimental Validation and Clinical Feasibility. Sci Rep 2021;11:12252.
